# Whole Brain and Brain Regional Coexpression Network Interactions Associated with Predisposition to Alcohol Consumption

**DOI:** 10.1371/journal.pone.0068878

**Published:** 2013-07-23

**Authors:** Lauren A. Vanderlinden, Laura M. Saba, Katerina Kechris, Michael F. Miles, Paula L. Hoffman, Boris Tabakoff

**Affiliations:** 1 Department of Pharmacology, University of Colorado School of Medicine, Aurora, Colorado, United States of America; 2 Department of Biostatistics and Informatics, University of Colorado School of Public Health, Aurora, Colorado, United States of America; 3 Departments of Pharmacology and Neurology and the Center of Study of Biological Complexity, Virginia Commonwealth University, Richmond, Virginia, United States of America; Boston University School of Medicine, United States of America

## Abstract

To identify brain transcriptional networks that may predispose an animal to consume alcohol, we used weighted gene coexpression network analysis (WGCNA). Candidate coexpression modules are those with an eigengene expression level that correlates significantly with the level of alcohol consumption across a panel of BXD recombinant inbred mouse strains, and that share a genomic region that regulates the module transcript expression levels (mQTL) with a genomic region that regulates alcohol consumption (bQTL). To address a controversy regarding utility of gene expression profiles from whole brain, vs specific brain regions, as indicators of the relationship of gene expression to phenotype, we compared candidate coexpression modules from whole brain gene expression data (gathered with Affymetrix 430 v2 arrays in the Colorado laboratories) and from gene expression data from 6 brain regions (nucleus accumbens (NA); prefrontal cortex (PFC); ventral tegmental area (VTA); striatum (ST); hippocampus (HP); cerebellum (CB)) available from GeneNetwork. The candidate modules were used to construct candidate eigengene networks across brain regions, resulting in three “meta-modules”, composed of candidate modules from two or more brain regions (NA, PFC, ST, VTA) and whole brain. To mitigate the potential influence of chromosomal location of transcripts and cis-eQTLs in linkage disequilibrium, we calculated a semi-partial correlation of the transcripts in the meta-modules with alcohol consumption conditional on the transcripts' cis-eQTLs. The function of transcripts that retained the correlation with the phenotype after correction for the strong genetic influence, implicates processes of protein metabolism in the ER and Golgi as influencing susceptibility to variation in alcohol consumption. Integration of these data with human GWAS provides further information on the function of polymorphisms associated with alcohol-related traits.

## Introduction

The concept of networks is critical to understanding biology at a systems level [1], [2], [3]. The availability of genome-wide measures of gene (transcript) expression levels provides the opportunity to identify gene coexpression networks, which have been reported to reflect biologically meaningful clustering of gene products [4], [5], [6] A further benefit of this approach is the identification of the genetic basis for regulation of the coexpression networks (genetics of gene expression), i.e., determination of the genetic markers or genomic regions that are associated with quantitative variation of transcript expression levels [7]. At the single gene level, the correlation of gene expression levels with a complex biological trait, combined with quantitative trait locus (QTL) analysis that identifies common genomic regions that regulate gene expression (eQTL) and the biological trait (bQTL), has been used by us and others to identify candidate genes for various complex phenotypes [8], [9], [10], [11], [12]. The same approach can be applied to transcriptional networks comprising gene coexpression modules. Such analysis allows for the description of genetically-regulated pathways that are associated with a complex phenotype, and also take gene-gene interactions into account [13], [14]. This approach has the potential to identify common signaling pathways that are associated with a trait in different populations, even if different individual genes/transcripts are associated with the trait in each population.

Controversy exists as to whether gene expression profiles from whole organs, or specific cells or regions of organs, provide better indicators of the relationship of measures of gene expression to a phenotype. Certainly, if one “refines” a phenotype to one clearly associated with a defined anatomical entity, e.g., left ventricular hypertrophy or absence seizures, or, on a cellular level, the release of a neurotransmitter such as GABA, it is absolutely rational to isolate the anatomical locus or cell type displaying the phenotype of interest for gene expression studies. Even within an anatomical structure, it is evident that one can discern organization of expressed RNA elements that is indicative of a particular cell type (e.g., neurons/astroglia/oligodendrocytes in brain [6]; or various cell types in liver, http://phenogen.ucdenver.edu) and thus, tease out the contribution of particular components of the whole structure to a phenotype. However, complex phenotypes are a result of genetic and environmental influences that usually reflect an array of networks that occur not only within a single tissue or organ, or a single region of a tissue or organ, but that interact between regions and between tissues and organs [15], [16].This is particularly relevant to complex (polygenic) phenotypes known to involve several organs (e.g., obesity or diabetes), or interactions between anatomically distinct parts of an organ such as heart or brain (e.g., heart failure or compulsive behavior). Recent gene expression-centered analysis of obesity has demonstrated the benefit of cross-organ analytical approaches to provide information about cross-organ communication (i.e., hypothalamus, white fat and liver) and coordinated cross-organ gene expression as a predisposing factor for obesity in mice [15]. Similarly, one can envision cross-regional networks within a complex anatomical structure, such as brain, that would contribute to a complex phenotype.

One highly investigated trait that has generated a number of studies using gene expression analysis is alcohol preference in mice. This phenotype is accepted to be polygenic, and QTL regions contributing to alcohol consumption/preference have been identified and replicated [17], [18], [19]. It is also accepted that this trait is a reflection of the coordinated function of a number of brain regions such as the brain “reward” system (ventral tegmental area (VTA), nucleus accumbens (NAc), striatum, etc.), executive areas of brain (frontal cortex areas), areas that control sensory systems (olfactory/taste), areas controlling reinforcement (hypothalamus), limbic areas (amygdala), areas involved in memory (hippocampus), and other areas [20]. It can be questioned whether measuring the endophenotypes of gene expression, or gene coexpression networks, in any particular region of brain is sufficient to generate insight into genomic determinants of this complex trait. Rather than attempting to generate insight into alcohol consumption behavior by studying gene expression/coexpression networks in only one area of brain [21], [22], or even studying several isolated areas, it may be more powerful to apply analytical techniques meant to provide evidence of transcriptional relationships across brain areas, so as to more thoroughly assess information exchange among the areas.

In the current study, we have used weighted gene coexpression network analysis (WGCNA) to identify and integrate gene coexpression networks in six selected brain regions, and in whole brain, to bring in transcript expression information from brain areas not directly sampled. Using a panel of BXD recombinant inbred (RI) mouse strains, we identified gene coexpression modules correlated with the predisposition to differences in alcohol consumption, and identified the genetic loci of control (QTLs) of these transcriptional networks. Candidate gene coexpression modules from each brain region and whole brain, in which the “module (m)QTL” overlapped a “behavioral (b)QTL” identified for alcohol drinking behavior, were used to construct second level networks across brain areas. This analysis produced “meta-modules” composed of candidate modules from two or more brain areas and whole brain that generate insight into the brain areas that contribute to predisposition to variation in the level of alcohol drinking, and the transcripts coordinately regulating this complex trait across several brain areas.

## Materials and Methods

### Phenotype Data

Data on alcohol consumption by BXD recombinant inbred (RI) strains were retrieved from GeneNetwork (www.genenetwork.org/). Two experiments involving BXD RI panels and alcohol consumption in the two-bottle choice (2BC) paradigm were used [18], [19]. These were the only two studies available that tested more than 15 BXD strains (Rodriguez et al., 1994 included 21 strains and Phillips et al., 1994 included 19 strains) and used a 2BC ethanol consumption measurement without prior exposure to ethanol. The Rodriguez et al. [19] data represent average daily alcohol consumption (g/kg) by males (50–70 days old), over a 15-day period of a two-bottle choice between 10% ethanol and tap water, whereas Phillips et al. [18] reported the average daily alcohol consumption (g/kg) by females (51 to 125 days old, average 87 days old) on day 2 and 4 of a 4-day period of access to 10% ethanol and tap water. Although alcohol consumption was measured in different sexes, the phenotypes across the BXD strains from these two studies have a significant correlation of 0.79 (p-value <0.001). It should be noted that phenotypic data collected on inbred strains remain stable over time, and, more specifically, Wahlsten and colleagues [23] showed that alcohol drinking behavior in 9 inbred strains (including the BXD parental strains, C57BL/6 and DBA/2) maintained the same rank order for over 40 years and across different laboratories.

### Whole Brain Gene Expression Measurements (Focus on Predisposition)

Gene expression data were generated in our laboratory in Colorado from whole brain tissue of naïve (non-alcohol-exposed) 70–94-day-old male mice using Affymetrix mouse whole genome oligonucleotide arrays (GeneChip Mouse Genome 430 v2.0 Array, Affymetrix, Santa Clara, CA). These data were obtained under protocols approved by the University of Colorado Anschutz Medical Campus Animal Care and Use Committee. Animals were euthanized according to the recommendations of the American Veterinary Medical Association guidelines on euthanasia. Transcript expression levels were measured in mice from 30 BXD RI strains (BXD1, BXD2, BXD5, BXD6, BXD8, BXD9, BXD11, BXD12, BXD13, BXD14, BXD15, BXD16, BXD18, BXD19, BXD21, BXD22, BXD23, BXD24, BXD27, BXD28, BXD29, BXD31, BXD32, BXD33, BXD34, BXD36, BXD38, BXD39, BXD40, BXD42) plus the 2 parental strains (C57BL/6J & DBA/2J) all purchased from the Jackson Laboratory. Four to seven mice per strain were used and RNA from each mouse was hybridized to a separate array. The methods are described in more detail in Tabakoff et al. [9], and all raw and processed data are available on http://phenogen.ucdenver.edu.

Prior to normalization, individual probes were removed if their nucleotide sequence did not uniquely map to a region in the mouse genome (NCBI 37/mm9) or if the probe contained a known single nucleotide polymorphism (SNP) between the two BXD parental strains based on data from whole-genome sequencing made available by the Sanger Institute [24]. Entire probesets were removed if less than 4 of the original 11 probes remained after this filter. Expression values were normalized and summarized into probesets using robust multichip analysis (RMA) [25]. The MAS5 algorithm [26] was used to evaluate if expression level measurements were above background (present, absent or marginal). If a probeset did not have at least one “present” call in any of the samples, the probeset was dropped from further analysis.

Data were thoroughly examined for batch effects related to processing. The microarrays were run over a year and a half period, resulting in 15 batches. Both batches and strains can contribute to non-random data distribution and a new method for removing batch effects, while retaining strain effects, was used (personal communication, Evan Johnson, Boston University). This method combines a simple rank test and a Bayesian hierarchical framework similar to the previously described empirical Bayes method [27].

Like the data on alcohol consumption, whole brain transcript expression levels have been shown by our laboratory to remain highly correlated over time (Figure S1).

### Brain Region Specific Expression Measurements

We obtained mRNA expression estimates from multiple brain areas of BXD RI mice by using publically available datasets through Gene Network (www.genenetwork.org). Datasets were included if the mice were either untreated or treated only with a saline injection, if the Affymetrix GeneChip Mouse Genome 430 v2.0 Array platform was used, and if expression values were normalized using RMA [25]. The brain areas that fit these criteria were cerebellum (GN accession# GN72), hippocampus (GN accession# GN110), nucleus accumbens (GN accession# GN156), prefrontal cortex (GN accession# GN135), striatum (GN accession# GN66) and ventral tegmental area (GN accession# GN228). All six brain areas, plus the whole brain, have data from 15 BXD RI strains in common (BXD5, BXD6, BXD9, BXD12, BXD15, BXD16, BXD19, BXD21, BXD27, BXD28, BXD31, BXD32, BXD33, BXD34, BXD38). Due to lack of information on present/absent calls for the datasets downloaded from GeneNetwork, and in order to allow for comparisons among gene expression networks identified in brain regions and whole brain [15], [16], [28], the brain regional datasets were filtered to contain the same probesets as were expressed above background in the whole brain data. To evaluate the validity of this procedure, we used raw data for gene expression from the ventral tegmental area of the BXD RI strains, that was obtained in the Miles laboratory. Analysis of these data showed that, depending on the strains used for the analysis, and the filtering criteria for “present” calls, 80–90% of probesets expressed above background in the ventral tegmental area dataset were also present in the whole brain dataset and, conversely, more than 90% of the probesets expressed above background in the whole brain dataset were also present in the ventral tegmental area dataset (Table S1 in file S1).

### Weighted Gene Coexpression Network Analysis (WGCNA)

WGCNA was performed separately on each of the 7 datasets (whole brain and brain regional data) to determine within-region coexpression networks. Expression data, after filtering for common probesets, from all available BXD RI strains for each dataset were utilized to create each network. The whole brain dataset consisted of 30 strains, cerebellum of 28 strains, hippocampus of 67 strains, nucleus accumbens of 34 strains, prefrontal cortex of 27 strains, striatum of 31 strains, and the ventral tegmental area of 35 strains. Data from parental strains were not used in statistical analyses to avoid confounding due to population structure. Strain mean expression values were used for all correlation measures.

An unsigned adjacency measure for each pair of transcripts was calculated by raising the absolute value of their Spearman correlation coefficient to a power of β. The proper power (β = 7) was determined by using the model-fitting index from Zhang and Horvath [29] with the whole brain dataset, and resulted in an approximately scale-free network. We applied the same power to the brain region specific networks. A scale-free network topology consists of a relatively few “hubs”, highly connected nodes (in our case, transcripts), and many other less connected nodes [29]. Most observed biological networks have been identified as scale-free, so it is reasonable to believe that the transcriptional networks should be as well [30], [31]. At this stage, we created unsigned networks, which allows grouping of probesets that are positively or negatively correlated with one another.

The adjacency measure was transformed into a topological overlap measure (TOM). This measure includes the direct relationship between two transcripts, i.e., their adjacency measure, and their indirect interactions based on their shared relationships with other genes in the network. A quantitative measure of indirect interactions between two transcripts is calculated by multiplying the adjacency measures of the two transcripts with a third transcript and summing the value across all other transcripts. The TOM is weighted in such a way that a value close to 1 for two genes signifies a high connectivity and co-expression, and will result in the genes being clustered within the same module.

We defined the distance between two genes as 1– TOM. Module detection was made using the TOM-based similarity measure coupled with average linkage hierarchical clustering and a dynamic tree cutting algorithm [32]. A distance criterion of 0.15 was implemented to distinguish individual modules. We chose to reduce the minimum module size from the default value of 30 to 5 to allow for identification of smaller modules, and therefore the inclusion of genes that would otherwise not be assigned to a module, without dramatically changing the composition of the larger modules. With smaller modules, functional enrichment analyses [33] are not applicable due to loss of power, but smaller modules allow for a more detailed knowledge-based investigation of the function of genes in the module.

### Identification and Characterization of Candidate Modules for Each Network

#### Summary Measurements

An eigengene, the first principal component of the module, was identified for each module and used as a summary of gene expression for the module. A hub gene was also identified for each module by determining the gene with the highest connectivity measurement within the module (i.e., sum of adjacencies with respect to other transcripts in the module).

#### Association with Phenotype

To identify modules associated with a predisposition to alcohol consumption, we calculated a Pearson correlation coefficient and its associated p-value between each eigengene and each alcohol consumption dataset from the 2 independent studies of 2BC alcohol consumption [18], [19]. We combined results from both consumption studies for each module using Fisher's method [34]. A false discovery rate (FDR) was implemented to account for multiple testing [35]. A module was considered associated if the FDR value was less than 0.05, or if the unadjusted Fisher's p-value was <0.01.

#### QTL Analysis

We identified expression quantitative trait loci (eQTLs) for individual transcripts and module quantitative trait loci (mQTLs) for individual modules (eigengenes) by performing marker regression QTL analysis using the single nucleotide polymorphism (SNP) dataset available via the Wellcome Trust (version 37, obtained from http://gscan.well.ox.ac.uk/gsBleadingEdge/mouse.snp.selector.cgi. Only SNPs with unique strain distribution patterns were used, based on the BXD RI strains available for each specific dataset. Empirical p-values were calculated using 1,000 permutations and considered significant if the resulting p-value was <0.05 [36]. Of interest are modules with a significant mQTL that overlaps a behavioral (b)QTL (i.e., alcohol consumption QTL), based on the rationale that if the expression level of genes within the module controls the variance of a behavior, the bQTL and mQTL should be localized within the same area of the genome [9].

We calculated bQTLs associated with alcohol consumption using behavioral data from Phillips et al. [18] and Rodriguez et al. [37] along with the SNP dataset described above (Wellcome Trust, version 37), and also using a marker regression algorithm. To be as inclusive as possible, we also considered bQTLs for alcohol consumption reported by Belknap and Atkins [17], which were based on a meta-analysis of alcohol preference studies of mapping populations derived from C57BL/6 and DBA/2 strains. The reported bQTL range in cM was converted to Mb using the mouse Map Converter (http:cgd.jax.org/mousemapconverter) [38]. All of the bQTLs are listed in Table S2 in file S1. Although it was not a criterion for distinction as a candidate module, we also examined each module to determine if a common eQTL location existed for the genes within the module. Genes were considered to be *cis* regulated if the eQTL was within 20 Kb of the gene [39].

#### Module Robustness

Robustness (quality) analysis was performed using module preservation statistics specifically for evaluating WGCNA modules [40]. We summarized robustness by reporting Z summary scores. The Z summary is a composite measure of 4 statistics related to density (i.e., highly connected nodes maintain that level of connectivity) and 3 statistics related to connectivity (i.e., connectivity pattern between specific genes is maintained). We used two methods to generate Z summary scores. First, to verify that our candidate modules were of high quality and not generated by chance, we examined reproducibility within a dataset. Using 100 bootstrap samples, we calculated the module preservation statistics for each bootstrap sample compared to the original dataset to generate a Z summary score of reproducibility. Z summary values between 2 and 10 are considered to be moderately preserved (reproducible), while those below 2 are considered not preserved, and those above 10 are considered strongly preserved [40]. Second, we compared the preservation of candidate modules between datasets (different brain areas). We used the brain area in which the module originated as a reference set, and the other brain regions as a test set for generating these Z scores.

### Eigengene Network

To determine how the candidate modules from all 6 brain regions and whole brain interact with each other, an eigengene network was constructed. All candidate module eigengenes were consolidated into one dataset and only the 15 strains that had expression data from 6 brain regions, and whole brain, were used. A signed network was created by performing WGCNA on this dataset; by keeping the direction of co-expression the same, we retain important biological function information [28]. In order to be conservative (i.e., to identify the most highly related modules), a distance (1– TOM) cut height of 0.5 was used to identify co-expressed candidate modules. We refer to these resulting co-expressed modules as meta-modules. To avoid examining a summary of a summary, we characterized the individual probesets within each candidate module that was a member of a meta-module. We calculated the connectivity for all probesets, identifying a hub gene, calculating a meta-mQTL by using the individual probesets to create a meta-eigengene, correlating the meta-mQTL with alcohol consumption and performing a knowledge-based search into the function of relevant genes. All meta-eigengenes were treated as individual variables and put into a multiple linear regression (PROC REG in SAS) to determine how much alcohol consumption variance is explained by the meta-modules for each 2BC study. The unadjusted R^2^ is reported.

### Adjustment for cis-eQTL effects on gene coexpression and phenotypic correlations

It has been pointed out that the expression levels of most genes with strong cis-eQTL tend to be highly correlated with other genes that have closely-linked (genetic position), strong cis-eQTLs [41]. This correlation could reflect a functional, biological relationship, but could also result from the fact that gene expression in a particular genetic region is controlled from closely liked genetic loci [15]. To investigate this latter possibility, we calculated a Pearson semi-partial correlation coefficient between each individual probeset within the candidate meta-modules and the phenotype, conditional on the most proximal marker to the genomic location of the individual probeset. We also calculated the partial correlation among probesets after accounting for the most proximal marker to the probesets. When the most proximal marker was not shared between two probesets, we calculated the residual expression values for each probeset after accounting for the most proximal SNP to that probeset and then correlated the residuals for a “modified” partial correlation.

### Integration of Mouse Data and Human GWAS Data

To integrate the results from the mouse transcriptome analysis with human GWAS results, human syntenic regions for the meta-mQTLs were determined. A 95% Bayesian credible interval was calculated for all meta-mQTLs and these intervals were input into the UCSC LinkOver tool to map the mouse (mm9) genome location to the human (hg19) genomic location (http://genome.ucsc.edu/cgi-bin/hgLiftOver). Various alcohol related phenotype GWAS [42], [43], [44], [45], [46] were examined for any associated SNPs residing within the syntenic region of the mQTLs. Knowledge-based searches on these syntenic regions were used for comparative genomics.

## Results

### Coexpression Modules from Whole Brain and Brain Regional Datasets

**Figure 1:**
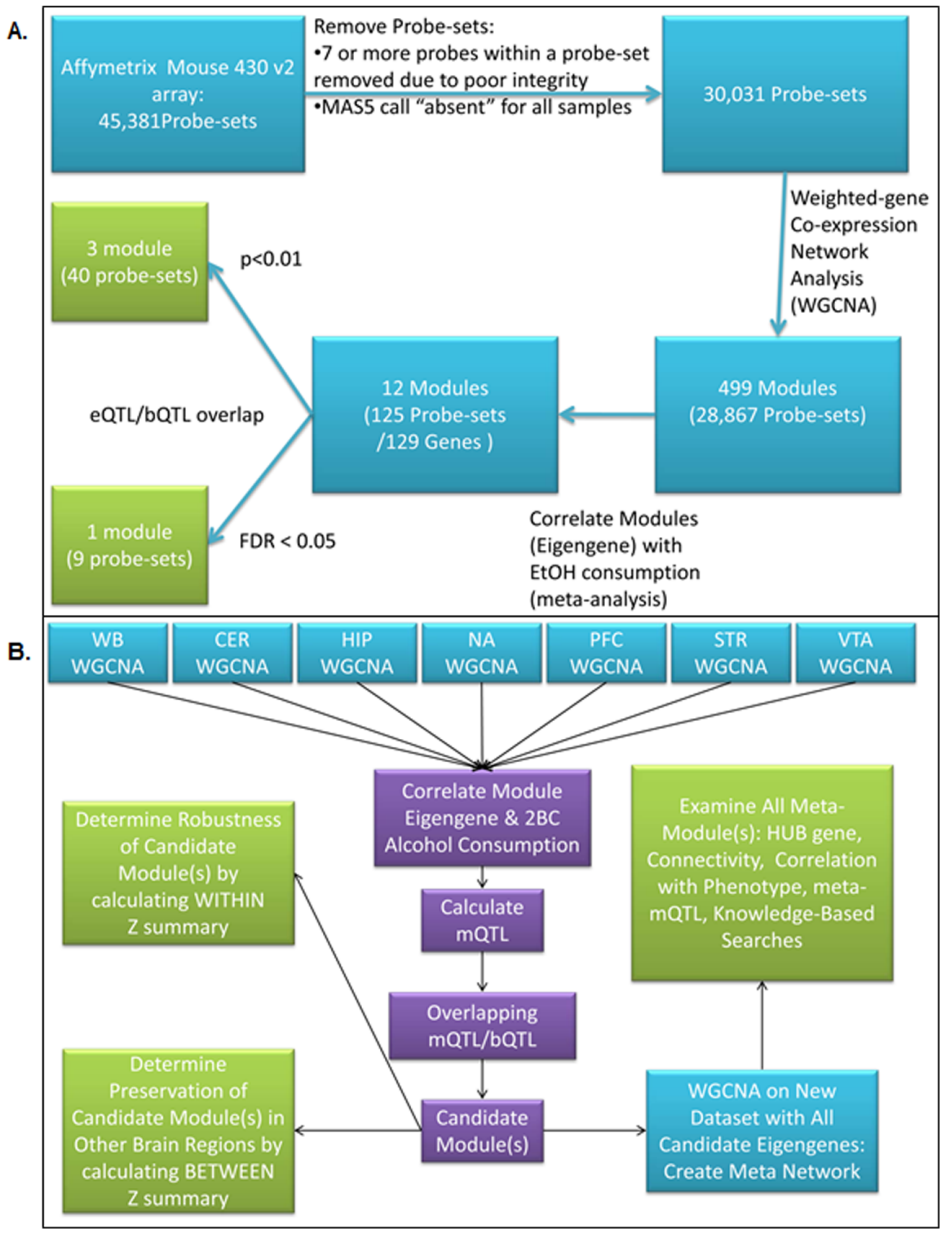
Flow Chart of Analysis Procedure for Whole Brain (A) and Brain Regional (B) Microarray Data. Whole brain microarray data were filtered for SNPs between C57BL/6 and DBA/2 mice, and for expression above background levels. The remaining probesets were subjected to WGCNA, and the resulting coexpression modules were filtered by correlation of eigengene with alcohol consumption data, followed by determination of overlap of mQTLs and alcohol bQTLs, to identify “candidate modules”. B. Microarray data for the indicated brain regions were obtained from GeneNetwork (www.genenetwork.org), and subjected to WGCNA (using the same probesets as were used for the whole brain data). Candidate modules were identified and characterized within each network, and were used to create an eigengene network that demonstrates gene coexpression within and between brain regions.

Of the 41,581 probesets in the whole brain dataset that were retained after masking, 30,031 probesets were detectable above background levels, and, as described in Methods, these probesets were used for WGCNA of whole brain and brain regional data (Figure 1). The characteristics of coexpression modules created from each dataset are shown in Table 1. The whole brain dataset contained the highest number of probesets that were included in coexpression modules, while the nucleus accumbens dataset contained the smallest number of probesets that were included in coexpression modules. However, the number of resultant nucleus accumbens modules exceeded the number calculated for whole brain (Table 1).

**Table 1:** WGCNA Network Summary

	Whole Brain	Cerebe llum	Hippo campus	Nucleus Accumbens	Prefrontal Cortex	Striatum	Ventral Tegmental Area
Number of BXD RI Strains On Which Network is Based	30	28	67	34	27	31	35
Number of Probesets Placed Into Modules	28,867	26,109	18,949	16,632	19,288	21,687	20,550
Number of Modules Identified	499	604	337	512	595	300	433
Minimum Module Size	5	5	5	5	5	5	5
Maximum Module Size	2,881	4,270	3,185	2,928	2,144	3,903	3,440
Median Module Size	9	8	8	9	8	9	9
**Candidate Modules**							
Number of Modules Significantly^1^ Correlated with Alcohol Consumption	12	2	5	19	7	9	12
Modules Significantly Correlated with Alcohol Consumption and with mQTL/bQTL overlap (Candidate Modules)	4	1	1	8	4	1	5
Minimum Proportion of Variance in Candidate Module(s) Captured by Eigengene	91%	98%	99%	88%	82%	97%	83%

1 Significant association with alcohol consumption is defined as FDR <0.05 or Fisher's unadjusted p-value <0.01 for the association between module eigengene and alcohol consumption (Rodriguez et al., 1994; Phillips et al., 1994).

### Characterization of Candidate Modules

“Candidate” modules were those with module eigengenes that were significantly (p<0.01) correlated with the phenotypic data on alcohol consumption by BXD RI mice in the 2BC paradigm [18], [37], and that had a statistically significant (p<0.001) module QTL (mQTL) that overlapped with a behavioral QTL (bQTL) for alcohol consumption (Figure 1A). A total of twenty-four modules derived from whole brain or brain regional data met these criteria (Table 1). Expression data from whole brain, nucleus accumbens (NA), prefrontal cortex (PFC), and ventral tegmental area (VTA) yielded the highest number of candidate modules. These networks are visualized in Figure S2. We also determined the amount of expression variance within a candidate module that was captured by the module eigengene (first principal component). As shown in Table 1, for each module, the corresponding eigengene captured at least 82% of the variance, indicating that the eigengenes can be used to represent the modules in further analyses.

**Figure 2:**
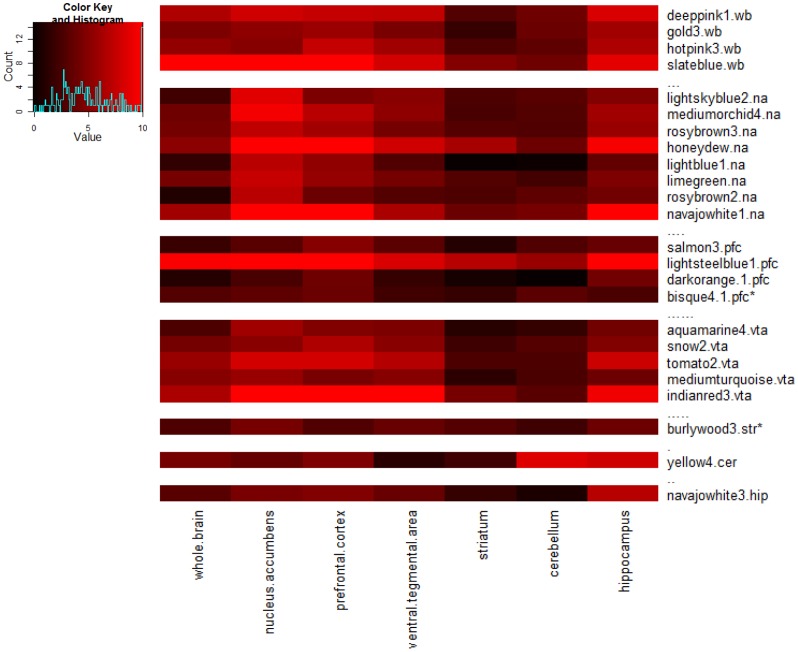
Reproducibility of Candidate Modules and Conservation of Candidate Modules across Brain Regions and Whole Brain. Conservation of candidate coexpression modules across individual brain regions and whole brain is represented by a Z summary score (color scale: 0 (black) to 10 (bright red)) (Langfelder et al., 2011; see text). In this graphic, Z summary scores above 10 are truncated to 10. The coexpression modules on the vertical axis are followed by an abbreviation indicating the network from which the module is derived: wb, whole brain; cer, cerebellum; hip, hippocampus; na, nucleus accumbens; pfc, prefrontal cortex; str, striatum; vta, ventral tegmental area. For each module, the Z summary score for conservation within each of the other datasets is shown. In addition, the average bootstrapped Z summary score is illustrated for the dataset from which the module was originally derived (represents reproducibility of candidate module in its original dataset). *Average Z summary score for reproducibility is within one SD of 2.

The characteristics of the candidate modules are shown in Table 2. In most of the candidate modules, the majority of the probesets have the same eQTL, which overlaps the mQTL region. As shown in Table S3 in file S1, within many of the modules, most transcripts have cis-eQTLs (i.e., the eQTL is within 20 Kb of the physical location of the transcript) [39]. Candidate module preservation mean Z summary scores ranged from 3.05 to 16.15. Two modules (bisque 4.1 (prefrontal cortex) and burlywood 3 (striatum)) had a lower interval boundary below 2 when the range of ± two standard deviations was taken into account. Therefore, the large majority of candidate modules are considered to be moderately to highly “reproducible” [40]. It is notable that several of the modules derived from whole brain data or from brain regional datasets have the same hub gene (most connected transcript) (e.g., *Scd5d* is the hub gene for the whole brain slateblue module, the NA honeydew module, the PFC lightsteelblue module and the VTA indianred3 module). This finding suggests similarity among modules from different brain regions, and we also used module preservation statistics to evaluate the conservation of candidate modules between whole brain and brain regional expression datasets. The results of this analysis are shown as the heatmap in Figure 2. According to this analysis, modules derived from whole brain data show the highest conservation, based on the Z-scores, in the expression data from NA, PFC, VTA and hippocampus.

**Table 2:** Characteristics of candidate modules associated with alcohol consumption.

Brain Region	Module	Number of Probesets	Correlation Rodriguez (p-value)	Correlation Phillips (p-value)	Fisher's p-value (FDR)	Shared eQTL Location : Chr (Mb)/probesets with shared eQTL	mQTL location: Chr (Mb)	mQTL LOD score (p- value)	Hub Gene (Gene Symbol)
Whole Brain	deeppink1	16	0.29 (0.126)	0.47 (0.008)	8.33E-03 (0.379)	4 (129.2–140.5)/16	4 (136.2)	19.8 (<0.001)	calcium/calmodulin-dependent protein kinase II inhibitor 1 Gene (Camk2n1)
Whole Brain	gold3	6	−0.37 (0.045)	−0.41 (0023)	8.06E-03 (0.379)	4 (154.9–155.4)/6	4 (155.2)	17.4 (<0.001)	guanine nucleotide binding protein (G protein), beta 1 (Gnb1)
Whole Brain	hotpink3	9	0.62 (<0.001)	0.53 (0.003)	1.23E-05 (0.006)	7 (121.8–127.2)/9	7 (124.4)	8.5 (<0.001)	ribosomal protein S15A (Rps15a)
Whole Brain	slateblue	18	0.35 (0.056)	0.46 (0.010)	4.91E-03 (0.345)	9 (41.5–54.8)/17	9 (41.5)	9.7 (<0.001)	sterol-C5-desaturase (fungal ERG3, delta-5-desaturase) homolog (S. cerevisae) (Sc5d/Sc5dl)
Cerebellum	yellow4	34	0.74 (0.006)	0.54 (0.056)	2.79E-03 (0.99)	N/A	13 (20.0)	4.1 (0.039)	myeloid/lymphoid or mixed-lineage leukemia 3 (Mll3)
Hippocampus	navajowhite3	9	0.48 (0.045)	0.76 (<0.001)	3.25E-04 (0.11)	2 (93.3)/8	2 (93.3)	18.7 (<0.001)	low density lipoprotein receptor-related protein 4 (Lrp4)
Nucelus Accumbens	lightskyblue2	12	−0.63 (0.007)	−0.79 (<0.001)	4.52E-05 (0.021)	2 (80.2–97.3)/12	2 (88.2)	18.3 (<0.001)	RIKEN cDNA 2810002D19 gene (2810002D19Rik)*
Nucelus Accumbens	mediumorchid4	14	−0.67 (0.003)	−0.73 (0.002)	8.48E-05 (0.021)	9 (56.7–62.2)/14	9 (56.7)	18.0 (<0.001)	neogenin (Neo1)
Nucelus Accumbens	rosybrown3	10	0.67 (0.003)	0.71 (0.003)	1.21E-04 (0.021)	9 (51.5–68.2)/10	9 (51.8)	16.1 (<0.001)	ELMO domain-containing protein 1 ( Elmod1)
Nucelus Accumbens	honeydew	27	0.36 (0.158)	0.72 (0.002)	3.33E-03 (0.182)	9 (35.8–41.5)/22	9 (41.5)	21.6 (<0.001)	sterol-C5-desaturase (fungal ERG3, delta-5-desaturase) homolog (S. cerevisae) (Sc5d)
Nucelus Accumbens	lightblue1	12	0.33 (0.196)	0.72 (0.002)	3.86E-03 (0.182)	4 (126.2–129.2)/12	4 (129.2)	21.4 (<0.001)	protein tyrosine phosphatase 4a2 (Ptp4a2)
Nucelus Accumbens	limegreen	10	−0.70 (0.002)	−0.31 (0.261)	4.05E-03 (0.182)	11 (101.5–108.1)/10	11 (101.5)	17.8 (<0.001)	LSM12 homolog (S. cerevisiae) (Lsm12)
Nucelus Accumbens	rosybrown2	9	0.50 (0.042)	0.63 (0.011)	4.18E-03 (0.182)	9 (35.0–48.0)/9	9 (48.0)	19.0 (<0.001)	asparagine-linked glycosylation 9 homolog (yeast, alpha 1,2 mannosyltransferase) (Alg9)
Nucelus Accumbens	navajowhite1	28	0.44 (0.077)	0.62 (0.014)	8.52E-03 (0.269)	4 (108.2–122.5)/28	4 (114.5)	22.1 (<0.001)	mediator of RNA polymerase II transcription, subunit 8 homolog (yeast) (Med8)
Prefrontal Cortex	salmon3	7	0.71 (0.001)	0.64 (0.010)	1.64E-04 (0.097)	7 (125.2–126.9)/5	7 (124.4)	10.0 (<0.001)	ribosomal protein S15A (Rps15a)
Prefrontal Cortex	lightsteelblue1	37	0.46 (0.063)	0.70 (0.004)	2.19E-03 (0.533)	9 (29.7–56.7)/29	9 (46.0)	16.4 (<0.001)	sterol-C5-desaturase (fungal ERG3, delta-5-desaturase) homolog (S. cerevisae) (Sc5d)
Prefrontal Cortex	darkorange.1	6	−0.58 (0.015)	−0.52 (0.049)	5.89E-03 (0.702)	2 (75.9–83.3)/5	2 (79.2)	9.2 (<0.001)	frizzled-related protein (Frzb)
Prefrontal Cortex	bisque4.1	6	0.64 (0.005)	0.35 (0.196)	8.37E-03 (0.714)	9 (28.1)/5	9 (28.1)	8.3 (0.016)	dentin matrix protein 1 (Dmp1)
Striatum	burlywood3	5	0.52 (0.020)	0.53 (0.025)	4.32E-03 (0.26)	7 (122.6–124.4)/5	7 (124.4)	15.6 (<0.001)	ribosomal protein S15A (Rps15a)
Ventral Tegmental Area	aquamarine4	6	0.67 (0.003)	0.81 (<0.001)	1.18E-05 (0.01)	9 (51.8–62.2)/6	9 (56.7)	14.7 (<0.001)	reticulocalbin 2 (Rcn2)
Ventral Tegmental Area	snow2	7	0.51 (0.037)	0.78 (0.001)	2.58E-04 (0.06)	9 (48.0)/7	9 (48.0)	22.6 (<0.001)	family with sequence similarity 55, member D (Fam55d)
Ventral Tegmental Area	tomato2	11	0.69 (0.002)	0.49 (0.061)	1.29E-03 (0.14)	7 (120.9–126.9)/10	7 (125.0)	19.2 (<0.001)	ribosomal protein S15A (Rps15a)
Ventral Tegmental Area	mediumturquoise	6	−0.67 (0.003)	−0.48 (0.069)	2.03E-03 (0.15)	11 (101.5–103.8)/6	11 (101.5)	14.0 (<0.001)	LSM12 homolog (S. cerevisiae) (Lsm12)
Ventral Tegmental Area	indianred3	21	0.30 (0.243)	0.71 (0.003)	6.35E-03 (0.26)	9 (34.9–50.5)/21	9 (41.5)	24.3 (<0.001)	sterol-C5-desaturase (fungal ERG3, delta-5-desaturase) homolog (S. cerevisae) (Sc5d)

Candidate modules from all whole brain and each brain regional network are shown. The first column depicts the network from which the candidate module was derived and the second column is the module name. The direction of the correlation is not reported as these are unsigned networks. N/A indicates there were no common eQTLs among the probesets. The mQTL location reports the chromosome and Mb location for the highest peak.

### Candidate Module Eigengene Network Analysis

**Figure 3:**
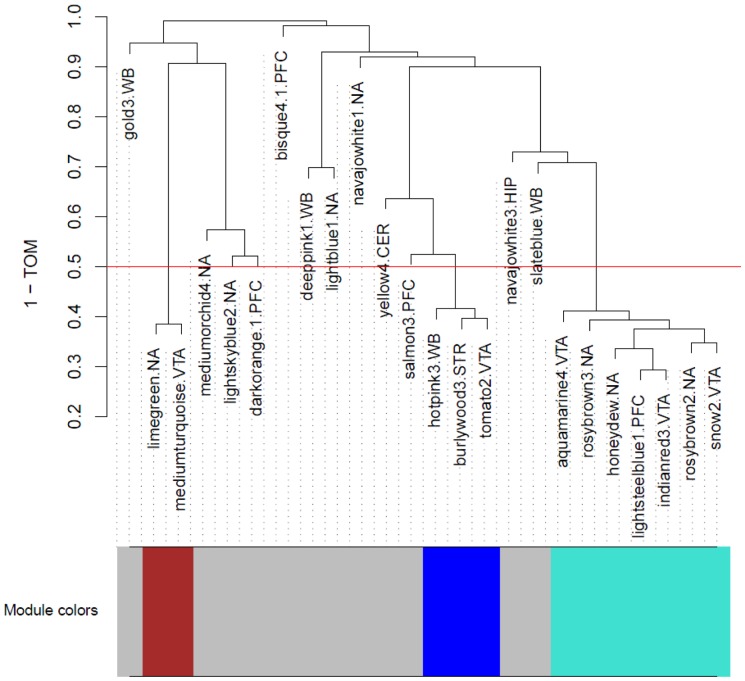
Eigengene Network. The eigengene network dendrogram was constructed based on a distance of (1-TOM) (see text). The red line ([1-TOM]  = 0.5) represents the criterion used for defining the meta-modules. Eigengenes colored grey were not assigned to a meta-module. The names of the candidate modules are followed by an abbreviation indicating the network from which these modules were derived: WB, whole brain; NA, nucleus accumbens; VTA, ventral tegmental area; PFC, prefrontal cortex, CER, cerebellum; HIP, hippocampus; STR, striatum.

In addition to identifying candidate coexpression modules from whole brain and brain regional expression data, we evaluated the higher order relationships among these modules, using a modification of the method described by Langfelder and Horvath [28]. In our analysis, we began with candidate modules from individual brain regions or whole brain, i.e., modules that were correlated with the phenotype of alcohol consumption, and met the added criterion of mQTL/bQTL overlap. All candidate modules were used for the eigengene network analysis. As a result, module relationships were not determined only within each brain region (each dataset), but relationships were also evaluated regardless of brain region network membership (i.e., candidate module relationships both within and across brain regions were determined). Figure 3 shows the meta-modules from the eigengene network that are correlated with alcohol consumption and that arise from several brain regions. The characteristics of the meta-modules are shown in Table 3, and the connectivity of the probesets that comprise the meta-modules is visualized in Figure S3. Each meta-module QTL is located on a different chromosome, and each meta-module includes common genes that are co-expressed in different brain regions and/or whole brain (Table S4 in file S1). These common transcripts represent the most highly connected genes (Figure S3) within modules in a particular brain area. Each meta-module also contains some less highly connected transcripts that are representative of only one brain region. It is of interest that, while the turquoise meta-module did not contain an eigengene from any of the whole brain candidate modules, we noted that the most connected genes in this meta-module were also identified within one or more of the whole brain candidate coexpression modules (Figure S3). In contrast, as an example, no genes from candidate hippocampal coexpression modules were included in any of the meta-modules. All three of the meta-modules accounted for 75 and 81% of the variance in alcohol consumption [18], [37].

**Table 3:** Meta-Module Characteristics.

Meta-Module	# Eigengenes (# probesets) (# unique genes)	Hub Gene (Gene Symbol)	Association with Alcohol Consumption^1^ (p-value)	mQTL^2^ Location: Chr (Mb)	mQTL^2^ LOD Score (p-value)	bQTL overlap
turquoise	7 (117) (65)	sterol-C5-desaturase (fungal ERG3, delta-5-desaturase) homolog (S. cerevisae) (Sc5d)	1.71E-03	9 (35.8)	7.1 (0.028)	Belknap & Atkins (et al., 2001)
blue	3 (29) (13)	ribosomal protein S15A Gene (Rps15a)	9.23E-04	7 (124.4)	5.2 (0.033)	Rodriguez (et al., 1994)
brown	2 (16) (13)	LSM12 homolog (S. cerevisiae) (Lsm12)	1.08E-02	11 (100.2)	3.9 (0.118)	Rodriguez (et al., 1994)

The size of each meta-module in the eigengene network is reported as number of eigengenes and number of probesets/genes. The hub gene is the highest connected gene of all probesets in the meta-module. ^1^Unadjusted Fisher's p-value is for association of meta-eigengene and alcohol consumption. ^2^The mQTL for the meta-module is reported as the location of the highest peak (Chr:Mb).

### Meta-Module Characterization

Most of the transcripts that comprise each meta-module are clustered in common chromosomal regions, and have proximal (cis) eQTLs that overlap with the meta-module QTL. In part, this is a result of our use of candidate modules for the eigengene WGCNA analysis, since a characteristic of a candidate module is that its mQTL overlaps with a behavioral QTL for alcohol consumption. The mQTL is calculated from the candidate module eigengene, and is a reflection of the eQTLs of the transcripts comprising the module. It has been suggested that chromosome-specific correlation patterns of gene expression result from gene expression traits controlled by closely linked genetic loci [15]. To investigate the correlation of the transcripts in the meta-modules with each other and with the alcohol consumption phenotype, while controlling for the effect of closely linked cis-eQTLs, we calculated the correlations of all transcripts in each meta-module conditional on the most proximal marker to the genomic location of the probeset. This analysis does not dismiss the relevance of the cis-eQTLs to the behavior or to transcript expression variation. Instead, the purpose of the analysis is to determine if the phenotype and transcript expression levels share any additional genetic (or environmental) determinants [41]. After this correction, sixteen individual probesets remained correlated with the phenotype (Fisher's combined P-value <0.10) (Table 4), and some of these probesets were also significantly (p<0.05) correlated with one another (Table S5 in file S1).

**Table 4:** Transcripts Significantly Correlated with Alcohol Consumption after Correction for cis-eQTL.

Module	Gene Symbol	Probeset	Rodriguez Correlation coefficient (p value)	Phillips Correlation coefficient (p value)	Fishers combined p-value
lightsteelblue1.pfc	Hyou1	1423291_s_at	0.89 (0.016)	0.92 (0.010)	0.002
honeydew.na	Hyou1	1423290_at	0.75 (0.089)	0.93 (0.008)	0.006
lightsteelblue1.pfc	Chpf2	1453846_at	0.91 (0.012)	0.68 (0.137)	0.012
rosybrown3.na	Arih1	1441022_at	−0.86 (0.030)	−0.63 (0.180)	0.033
rosybrown2.na	2310030G06Rik	1449357_at	−0.46 (0.354)	−0.89 (0.017)	0.038
mediumturquoise.vta	Lsm12	1427998_at	−0.87 (0.025)	−0.56 (0.249)	0.038
burlywood3.str	Rps15a	1457726_at	−0.51 (0.297)	−0.85 (0.033)	0.055
rosybrown3.na	Rcn2	1444248_at	−0.82 (0.045)	−0.59 (0.219)	0.056
honeydew.na	Fxyd2	1419378_a_at	−0.73 (0.097)	−0.73 (0.102)	0.056
lightsteelblue1.pfc	Alg9	1418844_at	−0.42 (0.401)	−0.84 (0.036)	0.075
rosybrown3.na	1700017B05Rik	1447063_at	0.52 (0.289)	0.81 (0.052)	0.078
lightsteelblue1.pfc	Ubash3b	1436805_at	0.69 (0.129)	0.71 (0.117)	0.079
tomato2.vta	Thumpd1	1436007_a_at	−0.80 (0.055)	−0.51 (0.296)	0.084
limegreen.na	Lsm12	1429509_at	0.73 (0.097)	0.64 (0.170)	0.084
lightsteelblue1.pfc	Sorl1	1460390_at	−0.64 (0.174)	−0.73 (0.096)	0.085
rosybrown3.na	Dis3l	1437737_at	−0.63 (0.176)	−0.71 (0.116)	0.100

Pearson semi-partial correlation coefficients were calculated between transcripts within meta-modules and the alcohol consumption phenotype, conditional on the most proximal marker to the genomic location of the individual probeset.

All of the transcripts in the meta-modules may contribute to the predisposition to consume alcohol, but those that remain correlated with the phenotype after correction for the cis-eQTL effect may be considered as the strongest candidates, and it is of interest to explore their function. We initially focused our attention on the transcripts that were found in the prefrontal cortex (turquoise meta-module). These transcripts are *Hyou1*, *Alg9*, *Chpf2*, *Ubash3b* and *Sorl1*. The products of these transcripts are associated with protein processing via the various compartments of the endoplasmic reticulum (ER) and protein degradation machinery. *Hyou1* (hypoxia up-regulated protein 1) (also called ORP150) is part of the ER chaperone network (chaperones of the heat shock protein family) that maintains protein folding [47], [48], and is induced by ER stress and hypoxia. This transcript was previously identified as a candidate gene for alcohol preference in whole brain and differential expression was validated by qRT-PCR [49], [50]. *Alg9* (asparagine-linked glycosylation 9, alpha-1, 2-mannosyltransferase homolog) is an ER enzyme that is involved in the synthesis of N-linked glycans [51]. *Alg9* and other related enzymes catalyze the synthesis of oligosaccharides that are transferred to the side chain amides of acceptor proteins. The N-glycans play a key role in quality control for protein folding in the ER, leading either to secretion of properly folded proteins or targeting of defective proteins for degradation [51]. *Chpf2* (chondroitin polymerizing factor 2, also called chondroitin synthase 3) is also an ER enzyme, which is involved in the synthesis of chondroitin sulfate, the polysaccharide (glycosaminoglycan) portion of several families of proteoglycans [52], [53]. The chondroitin chain is synthesized and modified (e.g., sulfated) in the ER and Golgi and attached glycosidically to serine in core proteins. There are numerous forms of proteoglycans [53], including those found in the brain extracellular matrix, which play important roles in neuronal plasticity [54]. *Ubash3b* (ubiquitin-associated and SH3 domain containing B) (also called Cbl-interacting protein Sts-1) has been implicated in protein degradation, specifically of the receptor tyrosine kinases, epidermal growth factor receptor (EGFR) and platelet-derived growth factor receptor (PDGFR) [55]. Ubash3B contains an SH3 domain that interacts with the ubiquitin ligase, Cbl, and a ubiquitin-associated domain that interacts with ubiquitin or a ubiquitin-protein complex. The interaction of Ubash3B with the EGFR complex inhibits receptor internalization (endocytosis) and blocks receptor degradation [55].

*Sorl1* (Sortilin-related receptor, L (DLR class) A repeats containing) is a transmembrane receptor that is found primarily in the trans-Golgi network (TGN) [56]. The TGN is a sorting compartment from which proteins are directed to secretory or degradative (endosomes or lysosomes) pathways. In particular, sortilin can bind to brain-derived neurotrophic factor (BDNF) and may direct BDNF into the regulated secretory pathway and/or to lysosomes [56].

In summary, the products of the transcripts from the prefrontal cortex that are correlated with the phenotype form a network related to protein processing in the ER and Golgi, including protein synthesis and degradation.

Many of the transcripts in the nucleus accumbens, VTA and striatum that are correlated with the phenotype are also linked to protein processing in the ER and Golgi, and to RNA metabolism. Transcripts in the nucleus accumbens include *Hyou1*, *Rcn2*, *Arih1*, *Dis3l* (turquoise meta-module) and *Lsm12* (brown meta-module). *Rcn2* (reticulocalbin 2, EF-hand calcium binding domain) codes for a protein that is a member of the CREC family of low affinity, Ca^2+^-binding proteins [57]. Its localization is restricted to the ER, where it may play a role in the protein secretory pathway, possibly as a chaperone [57], [58]. *Arih1* (ariadne homolog, ubiquitin-conjugating enzyme E2 binding protein; E3 ubiquitin protein ligase) is an enzyme associated with protein ubiquitination, a cascade that mediates regulated protein degradation and numerous other cellular processes including transcriptional regulation, protein trafficking and cellular signaling [59]. Protein ubiquitination involves transfer of ubiquitin between an activating enzyme (E1), a conjugating enzyme (E2) and a ligase (E3), which binds to E2 and enhances the transfer of ubiquitin from E2 to target [60]. The Arih1 protein is a member of the HECT family, a major class of ubiquitin ligases, and interacts with the E2 Ubch7 [61], [62], thought to be involved with cell proliferation and immune function. The product of *Dis3l* (Dis3 mitotic control homolog-like; Dis3-like exonuclease 1) is an exonuclease which, in human, is associated with the exosome, an exoribonuclease complex involved in the degradation and processing of a wide variety of RNAs [63], [64]. Other transcripts correlated with the phenotype in nucleus accumbens and VTA (brown meta-module) are *Lsm12*, *Thumpd1* (VTA, blue meta-module) and *Rps15a* (striatum, blue meta-module), all of which are involved with RNA metabolism. Lsm proteins, including Lsm12, accumulate in stress granules that are critical for regulation of translation and degradation of mRNA [65]. The Lsm12 protein has also been suggested to play a role in tRNA splicing and in methyl group transfer to tRNAs [66]. The product of *Thumpd1* (Thump domain containing 1) is a protein that contains an RNA binding domain that is fused to a methyltransferase that modifies tRNAs [67] *Rps15a* (ribosomal protein s15a) codes for a protein component of the 40S ribosomal subunit, which contributes to mRNA translation and protein synthesis. Another transcript correlated with the phenotype in the nucleus accumbens (turquoise meta-module) is *Fyxd2* (Fyxd domain containing ion transport regulator 2; Na^+^/K^+^ ATPase subunit gamma). The protein product of this transcript regulates the affinity of Na^+^/K^+^ ATPase for Na^+^ [68], and the activity of this enzyme is important for maintaining the cell membrane potential, which in turn affects protein trafficking processes [69].

### Integration with GWAS

One of the goals of the approach used here is to generate information on intermediate transcript expression pathways between phenotypes and genetic polymorphisms found to be associated with the phenotypes in genome-wide association studies. This is particularly important since many identified genetic polymorphisms do not reside in protein coding regions of genes, but in regulatory regions of the genome [70]. The human syntenic regions for the mouse meta-module QTL regions were compared with several recent GWAS for alcohol drinking and alcohol dependence. Table 5 shows the mouse chromosomal regions of the meta-module QTLs and the corresponding syntenic regions of the human genome. Several genetic polymorphisms (SNP or CNV) that were found to be associated with alcohol consumption parameters or alcohol dependence in humans are located within the corresponding regions of the mouse genome that regulate (mQTL) the gene co-expression modules that are associated with alcohol preference in mice. One of the human GWAS SNPs (rs7925049) is located within 5 Kb of a U6 snRNA, which is an important component of the spliceosome, and interacts with Lsm proteins [66], [71].

**Table 5:** Integration of Gene Expression and GWAS Data.

Study	Meta-Module	turquoise	blue	brown
	**95% CI mQTL: chr** **(Mb range)**	9 (29.7−56.7)	7 (124.4−127.1)	11 (98.3−111.0)
	**Human Syntenic Region(s): chr (Mb- range)**	11 (107.5−131.2)	16 (18.6−21.0) & 16 (17.0−17.7)	17 (37.9−68.3)
**Heath et al., 2011**	**SNP associated with alcohol dependence** **factor score**	**rs7925049: chr11 (122, 452,193) U6**		
	**SNP associated with 12-month weekly alcohol consumption**			rs6501422: chr17 (66,389,646)
	**SNP associated with DSM-IV alcohol dependence diagnosis in family-based analyses**	rs1785039: chr11 (127,900,579)		
**Edenberg et al., 2010**	**Replication of SNPs in** **GWAS of German** **Alcoholics (Table4)**			rs12603061: chr17 (64,812,198)
**Pei et al., 2012**	**CNV associated with regular alcohol drinking (p<0.05 in Discovery Sample Only)**			CNV2260: chr17 (39,532,869–39,536,674)
**Beirut et al., 2010**	**SNPs associated with** **alcohol dependence.** **(p <10?-6)**	rs10893366: chr11 (125,178,403) rs10893365: chr11 (125,176,437) rs 750338: chr11 (125,172,593)	rs9302534: chr16 (18,048,710)	
**Lind et al., 2010**	**Top 30 most significant** **SNPs associated with AD in** **Australian Population**	rs1784300: chr11 (118,679,629)		rs16947824: chr17 (62,501,505)

Regions of the human genome that are syntenic with the mouse meta-module QTLs (95% Bayesian credible intervals) were identified. The table lists several GWAS of alcohol-related phenotypes in humans that have significantly associated polymorphisms within these syntenic regions. SNP rs7925049 is located within 5 Kb of U6 snRNA, which is shown to have a relevant association with Lsm12 (brown meta-module). All other SNPs/CNV are either not located in/near a gene, or the genes are not found in any of the mouse meta-modules.

## Discussion

The brain has been envisioned to consist of anatomically and functionally related networks which evolved to both segregate and integrate information [72], [73], [74]. The network properties of brain, in many cases, are conserved in space and time, and neuroimaging information can be transformed to demonstrate consistent modularity, the existence of highly connected “hub” entities, and high efficiency of information transfer [75], [76]. Bullmore and Sporns [76] have utilized “graph theory” to demonstrate that brain functional networks, generated from MRI, EEG and MEG data, can span “multiple spatially distinct brain regions” and connote that the functional networks, rather than the isolated brain regions, provide the basis for the physiological function of brain and “mental representations”.

Network theory [77] has also been applied to global studies of gene expression [15], [56], [78] with the premise that the calculated networks can provide organizational information relevant to function at a cellular [78], organ [15], and organism [56] level. One of the popular network construction methods which have been applied to gene expression data is WGCNA [6], [32], [79], [80], [81], [82]. This approach can generate scale-free transcriptional networks consisting of modules, edges, and hubs [79], [83]. More recently, WGCNA has been applied to a higher level of organismal organization to discern cross-tissue relationships of gene expression and provide links between genetics, gene expression and phenotype [15], [16].

Most psychiatric phenotypes are complex (polygenic) traits that involve several anatomical regions of brain. Brain can be considered a multi-tissue organ, because of the anatomical organization of different cell types into regional nuclei (collection of cell bodies). The anatomical regions associated with an animal's predisposition to consume addictive substances are many [20].Certain publications have contended that benefit can be derived by studying gene expression in one or more areas of brain [22], while others have studied the relationship of whole brain gene expression levels to a phenotype such as ethanol preference [9], [10]. However, to date, there has not been a comparison of candidate gene networks for a complex trait that were identified within vs across brain regions. The strategy that we employed was focused on generating and utilizing gene coexpression network structure derived from mouse whole brain gene expression data, as well as data from six anatomically distinct areas of brain, to arrive at a global representation of gene expression network structure associated with the trait of alcohol preference.

In an attempt to ascertain whether there are relationships across brain areas between “candidate modules” identified in gene expression networks constructed from data for each brain area, meta-modules were constructed from all candidate modules in each brain area and whole brain using WGCNA. The analysis generated three meta-modules that can potentially indicate regulatory processes that encompass more than one brain region, and that reflect cross-regional signaling pathways associated with predisposition to alcohol consumption. Each of the meta-modules had candidate modules from more than one brain area, indicating a closer connectivity between candidate modules from different brain areas than certain modules within a brain area. Within a meta-module, certain candidate modules had the same hub gene. The mQTL location for each meta-module was within only one of the several bQTLs for alcohol consumption. These meta-modules contain candidate modules primarily from the NAc and VTA, areas which have been extensively linked with generating attention to rewarding/reinforcing situations [84] and especially to the availability of reinforcing/addictive substances [20], [85]. The other brain areas appearing in some of the meta-modules are the frontal cortex and striatum, as well as modules from analysis of whole brain. The striatum is in line of communication from the NAc with regard to the action necessary for obtaining alcohol “reward”, and the frontal cortex provides the “executive function” which dampens behavior that is generated in response to signaling through the NAc regarding the possibility of obtaining “reward”. Clearly, and maybe not surprisingly, our WGCNA analysis of gene expression in various brain areas related to the phenotype of alcohol consumption has focused our attention on anatomical areas previously reported to be involved in determining levels of alcohol consumption. What our data add, however, is that gene expression levels, when organized into modules and networks, can distinguish between brain areas more or less important to a phenotype, and the surprising result that modules correlated with alcohol consumption (and possibly other phenotypes) organize into meta-modules such that the overall control of most (if not all) transcripts included in a meta-module is from a segment of the genome that is identified as one bQTL (albeit the bQTLs identify a general region rather than an individual locus). Further analysis is needed to determine whether this characteristic of segregation of one meta-module to one bQTL is a general characteristic of gene expression in relation to any particular complex trait.

The analysis that brought us to the identification of candidate modules, and meta-modules, is based on the premise that transcript expression levels, or coexpression modules, that are correlated across the RI strains with the phenotype, and that have genomic regulatory regions in common with the regions that regulate the phenotype (e/mQTL/bQTL overlap), represent the strongest candidate genes/networks associated with the phenotypic trait [9], [10], [11], [86]. However, it is a concern that this analysis may generate expression level correlations based on the genomic location of genes within haplotype blocks, rather than providing insights into functional relationships that determine gene coexpression patterns. It has been reported that genes with strong cis-acting eQTLs are most highly correlated with other genes that have closely linked, strong cis-acting eQTLs [41], and therefore that chromosome-specific correlation patterns reflect this fact rather than representing biologically relevant coexpression patterns [15]. When we examine the transcripts within each candidate module and meta-module, we note that a large proportion of the transcripts are regulated by closely linked cis-eQTLs, suggesting that at least some of the transcripts in the meta-modules are correlated with one another, and with the phenotype, based on the cis-eQTL structure. When we calculated the correlations with alcohol consumption of the transcripts within the meta-modules conditional on the eQTL for each transcript, the transcripts that remain significantly correlated with the alcohol consumption phenotype may reflect the most biologically relevant relationships.

For the most part, these transcripts code for proteins that are associated with protein processing – synthesis (through tRNA and mRNA metabolism), folding (chaperone proteins) and trafficking and degradation (ubiquitination, endosomal/lysosomal trafficking). The localization of the products of many of the correlated transcripts is the ER and Golgi apparatus, where the synthesis and maturation of extracellular membrane proteins occur. Our network analysis is carried out on brains of ethanol-naive animals, and thus, generates insight into the systems that are associated with the predisposition to consume alcohol. The mutation or knockout of one protein associated with these systems, ubiquitin-specific peptidase 46 (Usp 46), has very recently been demonstrated to reduce ethanol preference in mice [87]. This study validates the involvement of the system related to protein processing and protein degradation as an *in vivo* modulator of the phenotype of alcohol consumption.

It is also of interest that ethanol exposure affects the systems that we have identified. For example, ethanol has been reported to induce ER stress in the brain (ER stress is a result of perturbation of ER function, e.g., by hypoxia, that results in accumulation of misfolded or unfolded proteins), and thereby induce the expression of chaperone proteins [88], [89], [90], [91]. Furthermore, exposure of neurons to ethanol affects the trafficking (neuronal membrane insertion and endocytosis) of (for example) GABA_A_ [92] and NMDA [93] receptors. Based on these findings, one may postulate that pre-alcohol consumption differences in the activity of protein processing pathways associated with the ER and Golgi machinery in particular brain regions contributes to the “sensation” that an individual experiences when alcohol is consumed, thereby influencing the amount of alcohol consumption. In other words, the function of the proteins generated from the transcripts that we have identified may provide each individual with a particular “set-point” that allows him or her to “sense” the effect of ethanol. It is of interest also that while many of the transcripts that we identified are correlated with one another within brain regions, there are also some (Table S5 in file S1) that are correlated across brain regions. These correlations suggest the possibility that certain processes are coordinated across connected regions.

The characteristics of data derived from WGCNA analysis of whole brain need to be considered in some detail since argument exists on whether whole brain transcriptome analysis is informative for relating transcript abundance to complex traits. We would expect that if whole brain were capturing all the information generated from data from each of the areas of brain, then the whole brain network generated by WGCNA under our constraints would contain more or the same number of modules, compared to any of the individual brain areas (this was not the case). Additionally, if the majority of the modular organization of gene expression in whole brain were simply an aggregate of modules from other brain areas with robust modules, there would be some expectation that modules from the whole brain network would be evident in each meta-module, since they would be replicates of modules evident in one of the other tested brain areas. We, however, note that there is no absolute identity in transcript membership between any two modules across the tested brain areas, and thus, although many modules have similarities (high Z scores) across brain areas, each area retains certain anatomically specific transcript membership within modules.

Is there a rationale, therefore, for measuring gene expression and analyzing data from whole brain? It should be noted that a whole brain module did segregate to the blue meta-module, and when one examines the transcripts included in this whole brain module (hotpink3), the transcripts encompass 67% of the transcripts in the tomato2 module from the VTA and 75% of the transcripts from the burlywood3 module from the striatum. There is also a whole brain module (slateblue) in proximity (relatedness distance) to the modules aggregating from three brain regions in the turquoise meta-module. The slateblue module contains 87% of genes within the turquoise meta-module (Figure S3). Thus, one can posit, that on the transcript level, data from whole brain can identify the major portion of transcripts that are associated with each other and with the phenotype of alcohol consumption in relevant brain areas. Since there are also modules in the whole brain transcriptome network that are correlated with alcohol consumption, with mQTLs overlapping alcohol consumption bQTLs, one can hypothesize that whole brain data may identify certain modules not captured in the networks generated from the six brain areas which were assessed (e.g., arising in other relevant brain areas). It is noteworthy, however, that the gene expression variance demonstrated by the transcripts contained in our three final meta-modules accounts for 82% of the variance in the behavioral data.

A further goal of this analysis is to provide information regarding an intermediate transcriptional network between GWAS and phenotype that could explain the influence of the genetic polymorphisms [86]. With this goal in mind, we determined regions of the human genome that are syntenic with the mQTLs for the meta-modules obtained in our analysis, and identified several genetic variants in those regions that have been associated with alcohol-related traits in GWAS. In particular, one SNP (rs7925049) that was significantly associated with alcohol dependence in the study of Heath et al. [42], was in the syntenic region of the mQTL for the meta-turquoise module. This SNP is close to a U6 snRNA, which is part of the spliceosome, and is thought to be crucial for the splicing reaction [94]. The U6 snRNP (ribonucleoprotein) also contains numerous protein components, and Lsm proteins (such as Lsm 12 in the VTA or nucleus accumbens) are particularly important for the mechanisms of the splicing reaction [71], [94]. It is possible that the GWAS SNP (or an associated polymorphism) affects the snRNA/Lsm protein interactions that influence transcript isoforms in brain. This integration of the human GWAS and the mouse transcriptome analyses is an example of potential insights into the genetic control of alcohol consumption and dependence that can be provided by cross-species analyses.

## Supporting Information

Figure S1 (PDF)

Figure S2 (PDF)

Figure S3 (PDF)

File S1 (DOC)
