# Supplementary material for: Whole Brain and Brain Regional Coexpression Network Interactions Associated with Predisposition to Alcohol Consumption
Source: PLoS One. 2013 Jul 23;8(7):e68878. doi: 10.1371/journal.pone.0068878 (PMC3720886; doi:10.1371/journal.pone.0068878)
Supplement: Figure S1 — (PDF) [file pone.0068878.s001.pdf]

Correlation plot of C57BL/6 Brain Expression Data over Time

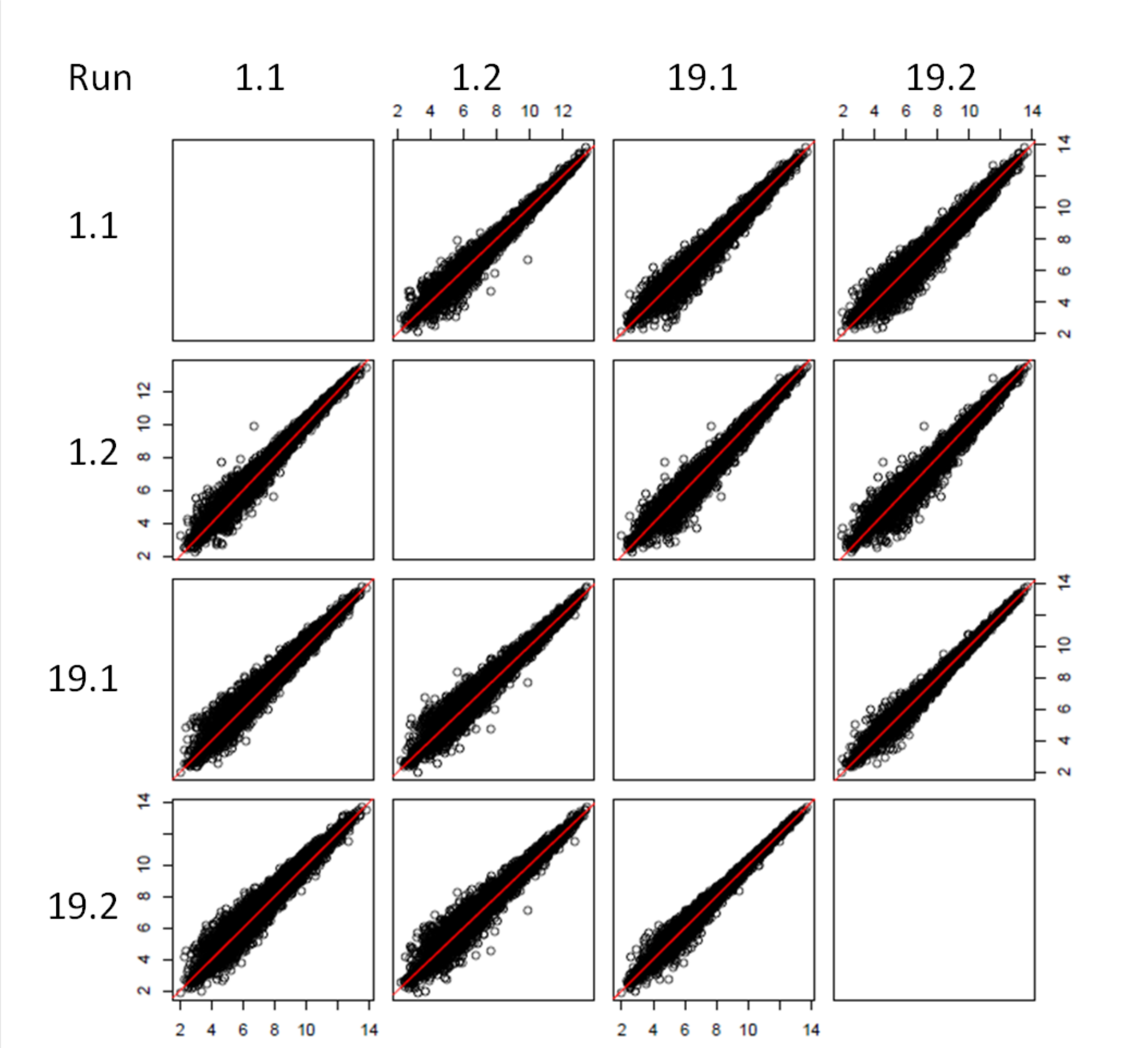

Figure S1 Correlation plot of C57BL/6 Brain Expression Data over Time
Affymetrix Mouse Exon 1.0 ST Arrays were used to measure transcript expression levels in C57BL/6 mouse whole brain. Scatter plots of core transcript cluster log2 (expression) levels obtained from microarrays processed in Feb 2010 (run 1.1 and 1.2) and June 2011 (run 19.1 and 19.2) are shown. The red line visualizes the Y = X. Pearson correlation coefficients were calculated for each combination of the 4 runs. All correlations are above 0.975, showing robust correlations of expression levels over time. In addition, gene expression intensities of mice in run1 and run19 were compared by one-way ANOVA (http://phenogen.ucdenver.edu/). No probeset was significantly different at FDR < 0.05.
