# Supplementary material for: Whole Brain and Brain Regional Coexpression Network Interactions Associated with Predisposition to Alcohol Consumption
Source: PLoS One. 2013 Jul 23;8(7):e68878. doi: 10.1371/journal.pone.0068878 (PMC3720886; doi:10.1371/journal.pone.0068878)
Supplement: Figure S2 — (PDF) [file pone.0068878.s002.pdf]

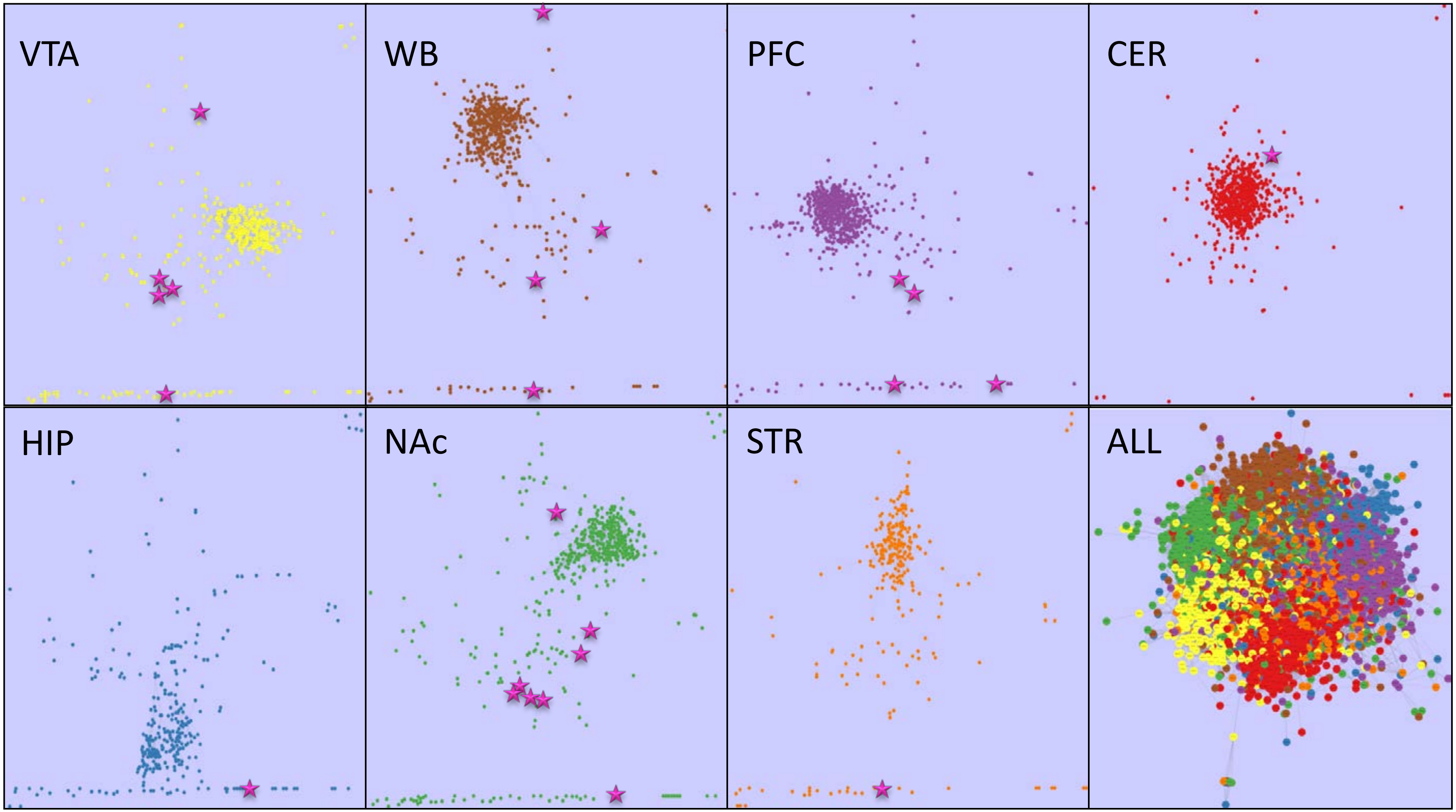


**Figure S2 Network Plots**

Plots visualize the module relationships to one another within each brain regional network or the whole brain network (using correlations between modules and the Cytoscape algorithm). Each node symbolizes a module and the Euclidean distance between nodes represents distance in correlation. Nodes with pink stars symbolize candidate modules within each network. Each panel represents a specific brain regional network (WB – whole brain; CER – cerebellum; HIP – hippocampus; NAc – nucleus accumbens; PFC – prefrontal cortex; STR – striatum; VTA – ventral tegmental area). The bottom-right panel labeled ALL represents all modules from all networks plotted together.
