# Supplementary material for: Whole Brain and Brain Regional Coexpression Network Interactions Associated with Predisposition to Alcohol Consumption
Source: PLoS One. 2013 Jul 23;8(7):e68878. doi: 10.1371/journal.pone.0068878 (PMC3720886; doi:10.1371/journal.pone.0068878)
Supplement: Figure S3 — (PDF) [file pone.0068878.s003.pdf]

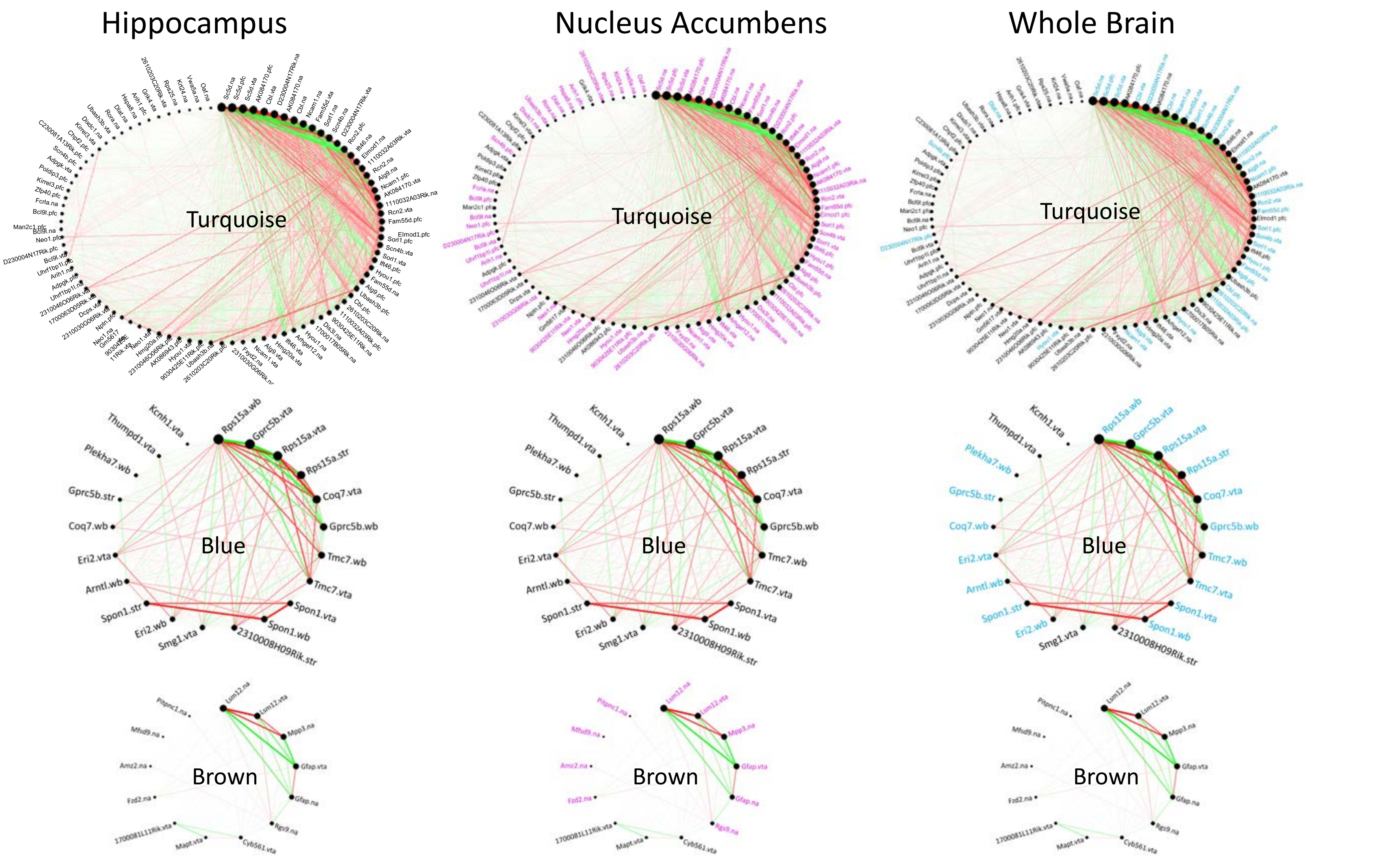

Figure S3 Connectivity and Brain Regional Localization of Transcripts in Meta-Modules
Plots visualize the adjacency between transcripts within each meta-module. The color and thickness of the lines represent the direction and strength of the correlation, respectively. Red represents positive, and green negative correlations. The size of the black dot for each transcript represents the overall connectivity measurement within the meta-module (the larger the dot, the more connected the gene). The gene name is followed by an abbreviation for the dataset from which the gene was identified (wb – whole brain; cer – cerebellum; hip – hippocampus; na – nucleus accumbens; pfc – prefrontal cortex; str – striatum; vta – ventral tegmental area). Pink highlighted gene names represent genes found in nucleus accumbens candidate modules, and blue highlighted gene names represent genes found in whole brain candidate modules. Genes from hippocampus candidate modules are not represented in any of the meta-modules.
