# Supplementary material for: Whole Brain and Brain Regional Coexpression Network Interactions Associated with Predisposition to Alcohol Consumption
Source: PLoS One. 2013 Jul 23;8(7):e68878. doi: 10.1371/journal.pone.0068878 (PMC3720886; doi:10.1371/journal.pone.0068878)
Supplement: File S1 — (DOC) [file pone.0068878.s004.doc]

| **Table S1 Comparison of Microarray Data from Whole Brain and Ventral Tegmental Area** | | | | | |
| --- | --- | --- | --- | --- | --- |
| **Using ALL Array Data Available (vta = 41 arrays, wb = 172 arrays)** | | | | | |
| **Filter Criteria (present in at least X% of samples)** | **# Whole Brain Probesets After Filter** | **# VTA Probesets After Filter** | **# Overlapping Probesets** | **% VTA Probeset the Whole Brain Captures** | **% Whole Brain Probeset the VTA Captures** |
| 1% | 30,031 | 29,856 | 27,674 | 92.7% | 92.2% |
| 10% | 23,654 | 26,281 | 22,306 | 84.9% | 94.3% |
| 50% | 19,370 | 22,316 | 18,074 | 81.0% | 93.3% |
| 75% | 17,188 | 20,473 | 16,032 | 78.3% | 93.3% |
| **Using Only Arrays that are Common Strains Among the 2 Datasets (21 strains + parentals, vta = 27 arrays, wb = 125 arrays)** | | | | | |
| **Filter Criteria (present in at least X% of samples)** | **# Whole Brain Probesets After Filter** | **# VTA Probesets After Filter** | **# Overlapping Probesets** | **% VTA Probeset the Whole Brain Captures** | **% Whole Brain Probeset the VTA Captures** |
| 1% | 27,836 | 29,270 | 26,090 | 89.1% | 93.7% |
| 10% | 23,709 | 26,682 | 22,453 | 84.2% | 94.7% |
| 50% | 19,350 | 22,321 | 18,054 | 80.9% | 93.3% |
| 75% | 17,172 | 20,337 | 15,969 | 78.5% | 93.0% |
| **Using Only Arrays that are Common Strains Among the 2 Datasets AND random pull/strain from WB to get same # arrays (21 strains + parentals, each dataset 23 arrays)** | | | | | |
| **Filter Criteria (present in at least X% of samples)** | **# Whole Brain Probesets After Filter** | **# VTA Probesets After Filter** | **# Overlapping Probesets** | **% VTA Probeset the Whole Brain Captures** | **% Whole Brain Probeset the VTA Captures** |
| 1% | 26,757 | 29,089 | 25,218 | 86.7% | 94.2% |
| 10% | 23,518 | 26,415 | 22,198 | 84.0% | 94.4% |
| 50% | 19,519 | 22,349 | 18,160 | 81.3% | 93.0% |
| 75% | 17,247 | 20,300 | 16,005 | 78.8% | 92.8% |
| **Parental Only, All Array data available (vta = 6 arrays, wb = 12 arrays)** | | | | | |
| **Filter Criteria (present in at least X% of samples)** | **# Whole Brain Probesets After Filter** | **# VTA Probesets After Filter** | **# Overlapping Probesets** | **% VTA Probeset the Whole Brain Captures** | **% Whole Brain Probeset the VTA Captures** |
| 1% | 23,448 | 26,390 | 21,884 | 82.9% | 93.3% |
| 10% | 22,047 | 26,390 | 20,953 | 79.4% | 95.0% |
| 50% | 18,768 | 21,768 | 17,401 | 79.9% | 92.7% |
| 75% | 17,023 | 20,578 | 15,905 | 77.3% | 93.4% |
| **Parental Only, Random Pull for WB so same # of Arrays (vta = 6 arrays, wb = 6 arrays)** | | | | | |
| **Filter Criteria (present in at least X% of samples)** | **# Whole Brain Probesets After Filter** | **# VTA Probesets After Filter** | **# Overlapping Probesets** | **% VTA Probeset the Whole Brain Captures** | **% Whole Brain Probeset the VTA Captures** |
| 1% | 22,775 | 26,390 | 21,485 | 81.4% | 94.3% |
| 10% | 22,775 | 26,390 | 21,485 | 81.4% | 94.3% |
| 50% | 18,700 | 21,768 | 17,341 | 79.7% | 92.7% |
| 75% | 17,616 | 20,578 | 16,292 | 79.2% | 92.5% |

| **Table S2 Calculated Alcohol Consumption bQTLs using WellcomeTrust build37 markers^a^** | | | | | |
| --- | --- | --- | --- | --- | --- |
| **Rodriguez: Male BXD Recombinant Inbred Panel** | | | | | |
| **Marker** | **Chromosome** | **Location (Mb)** | **LOD score** | **95% CI** | **20 Mb Range** |
| rs3713848 | 2 | 75.9 | 2.2 | (23.2 - 139.5) | 2 (69.2 - 89.2) |
| CEL-2_79237503 | 2 | 79.2 | 3.0 | (23.2 - 139.5) | 2 (69.2 - 89.2) |
| rs3667007 | 2 | 81.9 | 2.3 | (23.2 - 139.5) | 2 (69.2 - 89.2) |
| rs13479477 | 7 | 124.4 | 2.0 | (31.2 - 145.1) | 7 (116.3 - 136.3) |
| rs6241342 | 7 | 126.3 | 2.4 | (31.2 - 145.1) | 7 (116.3 - 136.3) |
| rs13481194 | 11 | 101.5 | 2.3 | (4.4 - 121.4) | 11 (91.5 - 111.5) |
| rs13481580 | 12 | 90.1 | 2.4 | (6.0 - 109.6) | 12 (80.1 - 100.1) |
| **Phillips: Female BXD Recombinant Inbred Panel** | | | | | |
| **Marker** | **Chromosome** | **Location (Mb)** | **LOD score** | **95% CI** | **20 Mb Range** |
| mCV23002990 | 2 | 81.9 | 2.6 | (81.9 - 88.2) | 2 (78.2 - 98.2) |
| rs6230107 | 2 | 88.2 | 3.2 | (81.9 - 88.2) | 2 (78.2 - 98.2) |
| UT_4_128.521481 | 4 | 129.2 | 2.5 | (3.6 - 155.2) | 4 (119.2 - 139.2) |
| rs3697583 | 4 | 132.4 | 2.1 | (3.6 - 155.2) | 4 (119.2 - 139.2) |
| CEL-8_7689226 | 8 | 7.7 | 2.0 | (7.7 - 126.2) | 8 (0 - 17.7) |
| rs3721858 | 13 | 12.2 | 2.1 | (12.2 - 120.1) | 13 (2.2 - 22.2) |
| **Meta-analysis of C57BL/6 and DBA/2 Derived Mice^b^** | | | |  |  |
|  | **Chromosome** | **Location (cM)** | **Location (Mb)^c^** |  |  |
|  | 2 | 9-49 | 11.7 - 81.7 |  |  |
|  | 3 | 48-83 | 108.7 - 160.2 |  |  |
|  | 4 | 59-75 | 125.2 - 141.5 |  |  |
|  | 9 | 9-61 | 22.2 - 111.3 |  |  |
| ^a^ bQTLs were re-calculated using the phenotype data of Rodriguez (1994) and Phillips (1994), and the Wellcome Trust build37. This calculation generates current positions and marker names for bQTLs associated with alcohol consumption. bQTL locations in Mb were taken directly from NCBI Mouse Genome Build 37. bQTL analyses were conducted in R (qtl package, Broman et. al., *Bioinformatics*, 19:889 (2003)). The 95% Bayesian Credible Interval and the 20 Mb range around the max LOD marker are reported for each bQTL. The 20 Mb range was used in determining overlapping eQTLs. | | | | | |
| ^b^bQTL locations in cM were taken from Belknap and Atkins (2001) and are based on a meta-analysis of several 2BC alcohol consumption studies using mapping populations derived from C57BL/6 and DBA/2 strains, i.e. BXD recombinant inbred panel, F2 crosses, selected lines, and backcrosses. Because these QTLs are based on a meta-analysis of many studies, the locations of the QTLs are reported as the range of peak LOD score locations from individual studies. | | | | | |
| ^c^cM positions were converted to Mb location in the NCBI Mouse Genome Build 37 using the Mouse Map Converter available through the Jackson Laboratory (http://cgd.jax.org/mousemapconverter/; Cox et al. *Genetics* 182:1335 (2009). Sex-averaged locations were used for conversion. | | | | | |

| **Table S3 Characteristics of Candidate Modules** | | | | | | | | | | | | | |
| --- | --- | --- | --- | --- | --- | --- | --- | --- | --- | --- | --- | --- | --- |
| **Cerebellum: yellow4** | | | | | | | | | | | | | |
| **Probeset** | **Gene Title** | | **Gene Symbol** | | **Connectivity** | | **Physcial Location: chr (Mb)** | | | **eQTL Location: chr (Mb)** | | **LOD score (p-value)** | |
| 1427150_at | myeloid/lymphoid or mixed-lineage leukemia 3 | | Mll3 | | 4.84 | | 5 (24.8) | | | 2 (139.3) | | 4.4 (0.015) | |
| 1438028_at | regulation of nuclear pre-mRNA domain containing 2 | | Rprd2 | | 4.04 | | 3 (95.6) | | | 13 (20.0) | | 4.1 (0.038) | |
| 1427310_at | bromodomain PHD finger transcription factor | | Bptf | | 3.57 | | 11 (106.9) | | | 2 (139.3) | | 4.3 (0.007) | |
| 1419867_a_at | ankyrin repeat and KH domain containing 1 | | Ankhd1 | | 3.53 | | 18 (36.8) | | | 17 (5.3) | | 4.1 (0.015) | |
| 1445273_at | DNA segment, Chr 10, ERATO Doi 638, expressed | | D10Ertd638e | | 3.25 | | 10 (66.6) | | | 13 (20.0) | | 3.7 (0.088) | |
| 1459869_x_at | pyruvate dehydrogenase phosphatase regulatory subunit | | Pdpr | | 3.09 | | 8 (113.7) | | | 13 (20.0) | | 3.6 (0.074) | |
| 1429009_at | small nuclear ribonucleoprotein 70 (U1) | | Snrnp70 | | 2.85 | | 7 (52.6) | | | 12 (40.2) | | 3.2 (0.169) | |
| 1455214_at | microphthalmia-associated transcription factor | | Mitf | | 2.83 | | 6 (98.0) | | | 17 (5.3) | | 4.9 (0.003) | |
| 1454051_at | family with sequence similarity 123, member A | | Fam123a | | 2.82 | | 14 (61.0) | | | 17 (5.3) | | 4.2 (0.018) | |
| 1417385_at | aminopeptidase puromycin sensitive | | Npepps | | 2.06 | | 11 (97.1) | | | 17 (32.1) | | 4.6 (0.012) | |
| 1451577_at | zinc finger and BTB domain containing 20 | | Zbtb20 | | 1.97 | | 16 (43.2) | | | 17 (31.8) | | 3.2 (0.154) | |
| 1432572_at | RIKEN cDNA 4931406H21 gene | | 4931406H21Rik | | 1.96 | | 14 (26.4) | | | 12 (40.2) | | 4.6 (0.019) | |
| 1457213_a_at | diacylglycerol kinase, eta | | Dgkh | | 1.77 | | 14 (79.0) | | | 17 (32.1) | | 4.3 (0.011) | |
| 1448909_a_at | mitochondrial ribosomal protein L39 | | Mrpl39 | | 1.41 | | 16 (84.7) | | | 12 (40.2) | | 2.9 (0.317) | |
| 1425686_at | CASP8 and FADD-like apoptosis regulator | | Cflar | | 1.38 | | 1 (58.8) | | | 2 (139.3) | | 3.3 (0.065) | |
| 1441728_at | sodium channel, voltage-gated, type I, alpha | | Scn1a | | 1.08 | | 2 (66.2) | | | 18 (58.6) | | 3.3 (0.166) | |
| 1443587_at | tetratricopeptide repeat domain 37 | | Ttc37 | | 1.05 | | 13 (76.3) | | | 13 (20.0) | | 4.3 (0.030) | |
| 1418562_at | splicing factor 3b, subunit 1 | | Sf3b1 | | 1.02 | | 1 (55.0) | | | 17 (5.3) | | 3.7 (0.017) | |
| 1453109_at | arylsulfatase K | | Arsk | | 0.88 | | 13 (76.2) | | | 17 (31.8) | | 3.5 (0.081) | |
| 1444021_at | sequestosome 1 | | Sqstm1 | | 0.84 | | 11 (50.0) | | | 13 (20.0) | | 4.2 (0.006) | |
| 1437129_at | RIKEN cDNA E330018D03 gene | | E330018D03Rik | | 0.79 | | 19 (45.8) | | | 14 (89.9) | | 4.1 (0.042) | |
| 1439612_at | calcium channel, voltage-dependent, N type, alpha 1B subunit | | Cacna1b | | 0.71 | | 2 (24.5) | | | X (133.1) | | 2.8 (0.241) | |
| 1460024_at | trinucleotide repeat containing 6b | | Tnrc6b | | 0.64 | | 15 (80.8) | | | 17 (60.5) | | 2.5 (0.381) | |
| 1441183_at | lysine (K)-specific demethylase 4B | | Kdm4b | | 0.63 | | 17 (56.5) | | | 17 (83.8) | | 2.0 (0.828) | |
| 1437230_at | potassium voltage-gated channel, shaker-related subfamily, member 1 | | Kcna1 | | 0.60 | | 6 (126.6) | | | 1 (83.9) | | 2.2 (0.646) | |
| 1441891_x_at | ELOVL family member 7, elongation of long chain fatty acids (yeast) | | Elovl7 | | 0.56 | | 13 (109.1) | | | 13 (107.1) | | 5.1 (0.006) | |
| 1455242_at | forkhead box P1 | | Foxp1 | | 0.52 | | 6 (98.9) | | | 10 (12.7) | | 4.1 (0.016) | |
| 1427896_at | suppressor of defective silencing 3 homolog (S. cerevisiae) | | Suds3 | | 0.49 | | 5 (117.5) | | | X (134.9) | | 2.8 (0.283) | |
| 1439600_at | NAD kinase | | Nadk | | 0.48 | | 4 (154.9) | | | 17 (89.4) | | 2.5 (0.545) | |
| 1448916_at | v-maf musculoaponeurotic fibrosarcoma oncogene family, protein G (avian) | | Mafg | | 0.46 | | 11 (120.5) | | | 2 (139.3) | | 3.0 (0.176) | |
| 1422444_at | integrin alpha 6 | | Itga6 | | 0.45 | | 2 (71.6) | | | 7 (77.3) | | 2.2 (0.690) | |
| 1434193_at | predicted gene 12942 | | Gm12942 | | 0.33 | | 4 (126.8) | | | 13 (20.5) | | 2.7 (0.271) | |
| 1451830_a_at | spectrin beta 2 | | Spnb2 | | 0.32 | | 11 (30.0) | | | X (132.7) | | 4.0 (0.054) | |
| 1424474_a_at | calcium/calmodulin-dependent protein kinase kinase 2, beta | | Camkk2 | | 0.32 | | 5 (123.2) | | | 16 (51.2) | | 2.2 (0.639) | |
| **Hippocampus: navajowhite3** | | | | | | | | | | | | | |
| **Probeset** | | **Gene Title** | | **Gene Symbol** | | **Connectivity** | | | **Physcial Location: chr (Mb)** | | **eQTL Location: chr (Mb)** | | **LOD score (p-value)** |
| 1426288_at | | low density lipoprotein receptor-related protein 4 | | Lrp4 | | 0.87 | | | 2 (91.3) | | 2 (93.3) | | 16.1 (<0.001) |
| 1425706_a_at | | damage specific DNA binding protein 2 | | Ddb2 | | 0.54 | | | 2 (91.1) | | 2 (93.3) | | 8.4 (<0.001) |
| 1439139_at | | DNA segment, Chr 2, ERATO Doi 640, expressed | | D2Ertd640e | | 0.46 | | | 2 (90.8) | | 2 (93.3) | | 8.5 (<0.001) |
| 1436680_s_at | | damage specific DNA binding protein 2 | | Ddb2 | | 0.44 | | | 2 (91.1) | | 2 (93.3) | | 6.3 (<0.001) |
| 1454965_at | | family with sequence similarity 171, member B | | Fam171b | | 0.41 | | | 2 (83.7) | | 2 (93.3) | | 7.3 (<0.001) |
| 1418038_s_at | | dual specificity phosphatase 19 | | Dusp19 | | 0.34 | | | 2 (80.5) | | 2 (93.5) | | 10.6 (<0.001) |
| 1452312_at | | RIKEN cDNA 2810002D19 gene | | 2810002D19Rik | | 0.29 | | | 2 (94.2) | | 9 (93.7) | | 21.7 (<0.001) |
| 1441001_at | | expressed sequence AI225934 | | AI225934 | | 0.26 | | | 2 (84.5) | | 2 (93.3) | | 7.7 (<0.001) |
| 1445621_at | | --- | | --- | | 0.18 | | | 2 (90.8) | | 2 (93.3) | | 10.3 (<0.001) |
| **Nucleus Accumbens: honeydew** | | | | | | | | | | | | | |
| **Probeset** | | **Gene Title** | | **Gene Symbol** | | **Connectivity** | | | **Physcial Location: chr (Mb)** | | **eQTL Location: chr (Mb)** | | **LOD score (p-value)** |
| 1451457_at | | sterol-C5-desaturase (fungal ERG3, delta-5-desaturase) homolog (S. cerevisae) | | Sc5d | | 5.94 | | | 9 (42.1) | | 9 (41.5) | | 17.5 (<0.001) |
| 1447047_at | | 12 days embryo eyeball cDNA, RIKEN full-length enriched library, clone:D230004N17 product | | AK084170 | | 5.49 | | | 9 (41.4) | | 9 (41.5) | | 13.0 (<0.001) |
| 1446608_at | | Casitas B-lineage lymphoma | | Cbl | | 5.49 | | | 9 (44.0) | | 9 (40.3) | | 12.2 (<0.001) |
| 1436467_at | | RIKEN cDNA D230004N17 gene /// hypothetical LOC100504017 | | D230004N17Rik /// LOC100504017 | | 5.18 | | | 9 (41.2) | | 9 (41.5) | | 11.4 (<0.001) |
| 1417474_at | | intraflagellar transport 46 homolog (Chlamydomonas) | | Ift46 | | 5.14 | | | 9 (44.6) | | 9 (41.5) | | 12.6 (<0.001) |
| 1426258_at | | sortilin-related receptor, LDLR class A repeats-containing | | Sorl1 | | 4.69 | | | 9 (41.8) | | 9 (41.5) | | 12.7 (<0.001) |
| 1434008_at | | sodium channel, type IV, beta | | Scn4b | | 4.62 | | | 9 (45.0) | | 9 (41.5) | | 12.0 (<0.001) |
| 1460390_at | | sortilin-related receptor, LDLR class A repeats-containing | | Sorl1 | | 4.50 | | | 9 (41.8) | | 9 (41.5) | | 8.2 (<0.001) |
| 1423290_at | | hypoxia up-regulated 1 | | Hyou1 | | 4.07 | | | 9 (44.2) | | 9 (41.5) | | 7.6 (<0.001) |
| 1448250_at | | RIKEN cDNA 9030425E11 gene | | 9030425E11Rik | | 3.67 | | | 9 (40.5) | | 9 (40.3) | | 11.5 (<0.001) |
| 1453003_at | | sortilin-related receptor, LDLR class A repeats-containing | | Sorl1 | | 3.53 | | | 8 (41.8) | | 9 (41.5) | | 8.1 (<0.001) |
| 1423291_s_at | | hypoxia up-regulated 1 | | Hyou1 | | 2.76 | | | 9 (44.2) | | 9 (40.9) | | 7.1 (0.002) |
| 1436805_at | | ubiquitin associated and SH3 domain containing, B | | Ubash3b | | 2.57 | | | 9 (40.8) | | 9 (40.3) | | 8.6 (<0.001) |
| 1448251_at | | RIKEN cDNA 9030425E11 gene | | 9030425E11Rik | | 2.38 | | | 9 (40.5) | | 9 (40.3) | | 8.0 (<0.001) |
| 1427437_at | | RIKEN cDNA 2610203C20 gene | | 2610203C20Rik | | 2.37 | | | 9 (41.4) | | 9 (41.5) | | 7.5 (<0.001) |
| 1456601_x_at | | FXYD domain-containing ion transport regulator 2 | | Fxyd2 | | 2.36 | | | 9 (45.2) | | 9 (41.5) | | 5.1 (0.006) |
| 1419378_a_at | | FXYD domain-containing ion transport regulator 2 | | Fxyd2 | | 1.93 | | | 9 (45.2) | | 9 (41.5) | | 5.2 (0.001) |
| 1453853_a_at | | Rho guanine nucleotide exchange factor (GEF) 12 | | Arhgef12 | | 1.60 | | | 9 (42.8) | | 9 (40.9) | | 5.8 (<0.001) |
| 1444855_at | | B-cell CLL/lymphoma 9-like protein | | Bcl9l | | 1.00 | | | 9 (44.3) | | 9 (41.5) | | 3.1 (0.142) |
| 1451913_a_at | | hypoxia up-regulated 1 | | Hyou1 | | 0.82 | | | 9 (44.2) | | 9 (41.5) | | 4.4 (0.009) |
| 1430978_at | | ribosomal protein S25 | | Rps25 | | 0.72 | | | 9 (44.2) | | 6 (138.4) | | 4.0 (0.058) |
| 1424709_at | | sterol-C5-desaturase (fungal ERG3, delta-5-desaturase) homolog (S. cerevisae) | | Sc5d | | 0.60 | | | 9 (42.1) | | 6 (90.3) | | 3.9 (0.029) |
| 1431182_at | | heat shock protein 8 /// hypothetical LOC624853 | | Hspa8 /// LOC624853 | | 0.55 | | | 9 (40.6) | | 9 (40.3) | | 4.2 (0.022) |
| 1425256_a_at | | DIX domain containing 1 | | Dixdc1 | | 0.42 | | | 9 (50.5) | | 6 (100.8) | | 2.3 (0.664) |
| 1426221_at | | von Willebrand factor A domain containing 5A | | Vwa5a | | 0.42 | | | 9 (38.5) | | 9 (35.8) | | 3.8 (0.020) |
| 1453327_at | | keratin 24 | | Krt24 | | 0.24 | | | 11 (99.1) | | 10 (9.3) | | 2.8 (<0.001) |
| 1457486_at | | OAF homolog (Drosophila) | | Oaf | | 0.24 | | | 9 (43.0) | | 6 (90.3) | | 2.4 (0.518) |
| **Nucleus Accumbens: lightskyblue2** | | | | | | | | | | | | | |
| **Probeset** | | **Gene Title** | | **Gene Symbol** | | **Connectivity** | | | **Physcial Location: chr (Mb)** | | **eQTL Location: chr (Mb)** | | **LOD score (p-value)** |
| 1445621_at | | --- | | --- | | 1.53 | | | 2 (90.8) | | 2 (88.2) | | 14.4 (<0.001) |
| 1441471_at | | RIKEN cDNA 2810002D19 gene | | 2810002D19Rik | | 1.16 | | | 2 (94.3) | | 2 (93.3) | | 9.3 (<0.001) |
| 1456759_at | | Leucine rich repeat containing 4C | | Lrrc4c | | 1.08 | | | 2 (97.3) | | 2 (94.4) | | 20.8 (<0.001) |
| 1439139_at | | DNA segment, Chr 2, ERATO Doi 640, expressed | | D2Ertd640e | | 0.93 | | | 2 (90.8) | | 2 (88.2) | | 7.7 (<0.001) |
| 1424654_at | | acid phosphatase 2, lysosomal | | Acp2 | | 0.92 | | | 2 (91.0) | | 2 (88.2) | | 9.9 (<0.001) |
| 1452312_at | | RIKEN cDNA 2810002D19 gene | | 2810002D19Rik | | 0.90 | | | 2 (94.2) | | 2 (93.3) | | 12.0 (<0.001) |
| 1442477_at | | autophagy/beclin 1 regulator 1 | | Ambra1 | | 0.80 | | | 2 (91.8) | | 2 (88.2) | | 9.5 (<0.001) |
| 1416401_at | | CD82 antigen | | Cd82 | | 0.69 | | | 2 (93.3) | | 2 (93.3) | | 8.1 (<0.001) |
| 1436350_at | | family with sequence similarity 171, member B | | Fam171b | | 0.58 | | | 2 (83.7) | | 2 (83.3) | | 11.4 (<0.001) |
| 1423612_at | | CLP1, cleavage and polyadenylation factor I subunit, homolog (S. cerevisiae) | | Clp1 | | 0.43 | | | 2 (84.6) | | 2 (83.3) | | 5.8 (<0.001) |
| 1418038_s_at | | dual specificity phosphatase 19 | | Dusp19 | | 0.39 | | | 2 (80.5) | | 2 (93.3) | | 4.7 (0.007) |
| 1426905_a_at | | DnaJ (Hsp40) homolog, subfamily C, member 10 | | Dnajc10 | | 0.39 | | | 2 (80.2) | | 2 (79.2) | | 6.6 (<0.001) |
| **Nucleus Accumbens: mediumorchid4** | | | | | | | | | | | | | |
| **Probeset** | | **Gene Title** | | **Gene Symbol** | | **Connectivity** | | **Physcial Location: chr (Mb)** | | | **eQTL Location: chr (Mb)** | | **LOD score**  **(p-value)** |
| 1447693_s_at | | neogenin | | Neo1 | | 2.74 | | 9 (58.7) | | | 9 (56.7) | | 9.8 (<0.001) |
| 1439784_at | | RIKEN cDNA 2310046O06 gene | | 2310046O06Rik | | 2.70 | | 9 (57.4) | | | 9 (56.7) | | 17.0 (<0.001) |
| 1441457_at | | neuroplastin | | Nptn | | 2.62 | | 9 (58.5) | | | 9 (56.7) | | 11.2 (<0.001) |
| 1440789_at | | neogenin | | Neo1 | | 2.36 | | 9 (58.8) | | | 9 (56.7) | | 9.0 (<0.001) |
| 1451079_at | | ADP-dependent glucokinase | | Adpgk | | 1.69 | | 9 (59.2) | | | 9 (56.7) | | 9.3 (<0.001) |
| 1459720_x_at | | --- | | --- | | 1.32 | | 10 (74.3) | | | 9 (62.2) | | 16.2 (<0.001) |
| 1434931_at | | neogenin | | Neo1 | | 1.24 | | 9 (58.7) | | | 9 (56.7) | | 8.9 (<0.001) |
| 1446240_at | | mitogen-activated protein kinase kinase 5 | | Map2k5 | | 1.18 | | 9 (63.1) | | | 9 (62.2) | | 12.2 (<0.001) |
| 1448153_at | | cytochrome c oxidase, subunit Va | | Cox5a | | 1.11 | | 9 (57.4) | | | 9 (56.7) | | 7.3 (<0.001) |
| 1438043_at | | high mobility group 20A | | Hmg20a | | 1.07 | | 9 (56.3) | | | 9 (56.7) | | 8.3 (<0.001) |
| 1448845_at | | ribonuclease P 25 subunit (human) | | Rpp25 | | 0.84 | | 9 (57.4) | | | 9 (56.7) | | 8.0 (<0.001) |
| 1423687_a_at | | mannosidase, alpha, class 2C, member 1 | | Man2c1 | | 0.73 | | 9 (57.0) | | | 9 (56.7) | | 5.7 (0.003) |
| 1418840_at | | programmed cell death 4 | | Pdcd4 | | 0.27 | | 19 (54.0) | | | 9 (56.7) | | 4.4 (0.016) |
| 1429203_at | | RIKEN cDNA 2410076I21 gene | | 2410076I21Rik | | 0.20 | | 9 (58.5) | | | 9 (56.7) | | 3.6 (0.030) |
|  | |  | |  | |  | |  | | |  | |  |
| **Nucleus Accumbens: rosybrown3** | | | | | | | | | | | | | |
| **Probeset** | | **Gene Title** | | **Gene Symbol** | | **Connectivity** | | **Physcial Location: chr (Mb)** | | | **eQTL Location: chr (Mb)** | | **LOD score (p-value)** |
| 1457223_at | | ELMO domain containing 1 | | Elmod1 | | 2.08 | | 9 (53.8) | | | 9 (51.8) | | 15.2 (<0.001) |
| 1444248_at | | reticulocalbin 2 | | Rcn2 | | 1.35 | | 9 (55.9) | | | 9 (51.8) | | 23.1 (<0.001) |
| 1428920_at | | high mobility group 20A | | Hmg20a | | 1.32 | | 9 (56.2) | | | 9 (51.8) | | 8.2 (<0.001) |
| 1459485_at | | neogenin | | Neo1 | | 1.30 | | 9 (58.8) | | | 9 (51.8) | | 7.6 (0.001) |
| 1437737_at | | DIS3 mitotic control homolog (S. cerevisiae)-like | | Dis3l | | 0.77 | | 9 (64.2) | | | 9 (51.8) | | 7.6 (<0.001) |
| 1443511_at | | RAR-related orphan receptor alpha | | Rora | | 0.75 | | 9 (68.8) | | | 9 (68.2) | | 7.2 (0.002) |
| 1423513_at | | UHRF1 (ICBP90) binding protein 1-like | | Uhrf1bp1l | | 0.65 | | 9 (58.7) | | | 9 (56.7) | | 7.9 (<0.001) |
| 1441181_at | | RAR-related orphan receptor alpha | | Rora | | 0.62 | | 9 (68.8) | | | 9 (68.2) | | 14.8 (<0.001) |
| 1447063_at | | RIKEN cDNA 1700017B05 gene | | 1700017B05Rik | | 0.48 | | 9 (57.1) | | | 9 (51.5) | | 6.5 (<0.001) |
| 1441022_at | | ariadne ubiquitin-conjugating enzyme E2 binding protein homolog 1 (Drosophila) | | Arih1 | | 0.37 | | 9 (59.2) | | | 9 (51.5) | | 5.5 (0.001) |
| **Nucleus Accumbens: lightblue1** | | | | | | | | | | | | | |
| **Probeset** | | **Gene Title** | | **Gene Symbol** | | **Connectivity** | | **Physcial Location: chr (Mb)** | | | **eQTL Location: chr (Mb)** | | **LOD score (p-value)** |
| 1420613_at | | protein tyrosine phosphatase 4a2 | | Ptp4a2 | | 2.23 | | 4 (129.5) | | | 4 (129.2) | | 19.0 (<0.001) |
| 1425054_a_at | | RIKEN cDNA 2510006D16 gene | | 2510006D16Rik | | 1.93 | | 4 (129.3) | | | 4 (129.2) | | 14.5 (<0.001) |
| 1440515_at | | doublecortin domain containing 2b /// doublecortin domain-containing protein 2B-like | | Dcdc2b /// LOC100504835 | | 1.92 | | 4 (129.3) | | | 4 (129.2) | | 12.1 (<0.001) |
| 1444714_at | | doublecortin domain containing 2b | | Dcdc2b | | 1.40 | | 4 (129.3) | | | 4 (129.2) | | 11.1 (<0.001) |
| 1415763_a_at | | RIKEN cDNA 2510006D16 gene /// hypothetical LOC100505293 | | 2510006D16Rik /// LOC100505293 | | 1.21 | | 4 (129.3) | | | 4 (129.2) | | 8.3 (<0.001) |
| 1444188_at | | --- | | --- | | 1.17 | | 4 (129.5) | | | 4 (129.2) | | 8.9 (<0.001) |
| 1440434_at | | protein tyrosine phosphatase 4a2 | | Ptp4a2 | | 0.83 | | 4 (129.5) | | | 4 (129.2) | | 7.4 (0.002) |
| 1434157_at | | taxilin alpha | | Txlna | | 0.82 | | 4 (129.3) | | | 4 (129.2) | | 6.3 (<0.001) |
| 1444377_at | | proteasome (prosome, macropain) subunit, beta type 2 | | Psmb2 | | 0.72 | | 4 (126.4) | | | 4 (126.2) | | 13.6 (<0.001) |
| 1446474_at | | histone deacetylase 1 | | Hdac1 | | 0.34 | | 4 (129.2) | | | 4 (129.2) | | 4.2 (0.023) |
| 1442678_at | | coiled coil domain containing 28B | | Ccdc28b | | 0.30 | | 4 (129.3) | | | 4 (129.2) | | 4.1 (0.020) |
| 1418630_at | | KH domain containing, RNA binding, signal transduction associated 1 | | Khdrbs1 | | 0.20 | | 4 (129.4) | | | 4 (129.2) | | 3.4 (0.039) |
| **Nucleus Accumbens: limegreen** | | | | | | | | | | | | | |
| **Probeset** | | **Gene Title** | | **Gene Symbol** | | **Connectivity** | | **Physcial Location: chr (Mb)** | | | **eQTL Location: chr (Mb)** | | **LOD score (p-value)** |
| 1429509_at | | LSM12 homolog (S. cerevisiae) | | Lsm12 | | 2.08 | | 11 (102.0) | | | 11 (101.5) | | 19.6 (<0.001) |
| 1427998_at | | LSM12 homolog (S. cerevisiae) | | Lsm12 | | 1.96 | | 11 (102.0) | | | 11 (101.5) | | 16.9 (<0.001) |
| 1419077_at | | membrane protein, palmitoylated 3 (MAGUK p55 subfamily member 3) | | Mpp3 | | 1.56 | | 11 (101.9) | | | 11 (101.5) | | 14.0 (<0.001) |
| 1440142_s_at | | glial fibrillary acidic protein | | Gfap | | 1.44 | | 11 (102.8) | | | 11 (101.5) | | 10.3 (<0.001) |
| 1439635_at | | regulator of G-protein signaling 9 | | Rgs9 | | 0.97 | | 11 (109.1) | | | 11 (108.1) | | 11.0 (<0.001) |
| 1434964_at | | archaelysin family metallopeptidase 2 | | Amz2 | | 0.67 | | 11 (109.3) | | | 11 (106.5) | | 9.8 (<0.001) |
| 1418534_at | | frizzled homolog 2 (Drosophila) | | Fzd2 | | 0.43 | | 11 (102.5) | | | 11 (101.5) | | 7.4 (<0.001) |
| 1417241_at | | archaelysin family metallopeptidase 2 | | Amz2 | | 0.29 | | 11 (109.3) | | | 11 (108.1) | | 7.3 (<0.001) |
| 1460619_at | | major facilitator superfamily domain containing 9 | | Mfsd9 | | 0.24 | | 1 (40.8) | | | 11 (108.1) | | 5.7 (<0.001) |
| 1428025_s_at | | phosphatidylinositol transfer protein, cytoplasmic 1 | | Pitpnc1 | | 0.23 | | 11 (107.1) | | | 11 (106.5) | | 5.2 (0.007) |
| **Nucleus Accumbens: rosybrown2** | | | | | | | | | | | | | |
| **Probeset** | | **Gene Title** | | **Gene Symbol** | | **Connectivity** | | **Physcial Location: chr (Mb)** | | | **eQTL Location: chr (Mb)** | | **LOD score (p-value)** |
| 1418844_at | | asparagine-linked glycosylation 9 homolog (yeast, alpha 1,2 mannosyltransferase) | | Alg9 | | 1.96 | | 9 (50.6) | | | 9 (48.0) | | 13.1 (<0.001) |
| 1417211_a_at | | RIKEN cDNA 1110032A03 gene | | 1110032A03Rik | | 1.86 | | 9 (50.6) | | | 9 (48.0) | | 13.1 (<0.001) |
| 1426865_a_at | | neural cell adhesion molecule 1 | | Ncam1 | | 1.83 | | 9 (49.3) | | | 9 (48.0) | | 16.4 (<0.001) |
| 1456111_at | | family with sequence similarity 55, member D | | Fam55d | | 1.81 | | 9 (48.2) | | | 9 (48.0) | | 11.8 (<0.001) |
| 1442680_at | | neural cell adhesion molecule 1 | | Ncam1 | | 1.69 | | 9 (49.5) | | | 9 (48.0) | | 16.2 (<0.001) |
| 1449357_at | | RIKEN cDNA 2310030G06 gene | | 2310030G06Rik | | 1.16 | | 9 (50.5) | | | 9 (48.0) | | 6.8 (<0.001) |
| 1443018_at | | neural cell adhesion molecule 1 | | Ncam1 | | 0.72 | | 9 (49.5) | | | 9 (48.0) | | 6.7 (<0.001) |
| 1426265_x_at | | dihydrolipoamide S-acetyltransferase (E2 component of pyruvate dehydrogenase complex) | | Dlat | | 0.24 | | 9 (50.4) | | | 9 (48.0) | | 3.3 (0.079) |
| 1419907_s_at | | Fc receptor-like A | | Fcrla | | 0.15 | | 1 (172.8) | | | 9 (35.0) | | 4.3 (0.024) |
| **Nucleus Accumbens: navajowhite1** | | | | | | | | | | | | | |
| **Probeset** | | **Gene Title** | | **Gene Symbol** | | **Connectivity** | | **Physcial Location: chr (Mb)** | | | **eQTL Location: chr (Mb)** | | **LOD score (p-value)** |
| 1442417_at | | mediator of RNA polymerase II transcription, subunit 8 homolog (yeast) | | Med8 | | 5.08 | | 4 (118.1) | | | 4 (114.5) | | 16.3 (<0.001) |
| 1442124_at | | expressed sequence AU022252 | | AU022252 | | 4.62 | | 4 (118.9) | | | 4 (114.5) | | 18.5 (<0.001) |
| 1445670_at | | --- | | --- | | 4.61 | | 4 (116.2) | | | 4 (114.5) | | 21.8 (<0.001) |
| 1439898_at | | EBNA1 binding protein 2 | | Ebna1bp2 | | 4.04 | | 4 (118.3) | | | 4 (114.5) | | 12.8 (<0.001) |
| 1433875_at | | RIKEN cDNA 4732418C07 gene | | 4732418C07Rik | | 3.95 | | 4 (115.4) | | | 4 (114.5) | | 15.4 (<0.001) |
| 1457668_x_at | | DNA segment, Chr 4, ERATO Doi 617, expressed | | D4Ertd617e | | 3.82 | | 4 (118.3) | | | 4 (114.5) | | 13.3 (<0.001) |
| 1441573_at | | Sex comb on midleg homolog 1 | | Scmh1 | | 3.55 | | 4 (120.1) | | | 4 (120.1) | | 24.8 (<0.001) |
| 1455378_at | | ribosomal modification protein rimK-like family member A | | Rimkla | | 2.93 | | 4 (119.1) | | | 4 (114.5) | | 8.8 (<0.001) |
| 1417461_at | | CAP, adenylate cyclase-associated protein 1 (yeast) | | Cap1 | | 2.42 | | 4 (122.5) | | | 4 (122.5) | | 29.8 (<0.001) |
| 1417462_at | | CAP, adenylate cyclase-associated protein 1 (yeast) | | Cap1 | | 2.41 | | 4 (122.5) | | | 4 (122.5) | | 31.9 (<0.001) |
| 1439499_at | | expressed sequence AA415398 | | AA415398 | | 2.30 | | 4 (119.2) | | | 4 (114.5) | | 8.8 (<0.001) |
| 1430593_at | | coiled-coil domain containing 30 | | Ccdc30 | | 1.94 | | 4 (119.0) | | | 4 (114.5) | | 7.7 (<0.001) |
| 1434524_at | | eukaryotic translation initiation factor 2B, subunit 3 | | Eif2b3 | | 1.76 | | 4 (116.7) | | | 4 (114.5) | | 7.1 (<0.001) |
| 1423073_at | | cytidine monophosphate (UMP-CMP) kinase 1 | | Cmpk1 | | 1.44 | | 4 (114.6) | | | 4 (114.5) | | 7.1 (<0.001) |
| 1429134_at | | human immunodeficiency virus type I enhancer binding protein 3 | | Hivep3 | | 1.36 | | 4 (119.4) | | | 4 (114.5) | | 5.6 (0.003) |
| 1446288_at | | expressed sequence C78692 | | C78692 | | 1.17 | | 4 (120.5) | | | 4 (120.1) | | 8.0 (<0.001) |
| 1454259_s_at | | ATP/GTP binding protein-like 4 | | Agbl4 | | 1.13 | | 4 (110.1) | | | 4 (111.0) | | 5.9 (0.002) |
| 1431423_a_at | | mediator of RNA polymerase II transcription, subunit 8 homolog (yeast) | | Med8 | | 1.08 | | 4 (118.1) | | | 4 (114.0) | | 7.6 (<0.001) |
| 1458879_at | | expressed sequence C76798 | | C76798 | | 0.95 | | 4 (123.6) | | | 4 (122.5) | | 7.8 (<0.001) |
| 1447164_at | | zinc finger protein Rlf | | Rlf | | 0.88 | | 4 (120.9) | | | 4 (121.1) | | 9.0 (<0.001) |
| 1421771_a_at | | IAP promoted placental gene | | Ipp | | 0.86 | | 4 (116.2) | | | 4 (114.5) | | 5.6 (<0.001) |
| 1446932_at | | --- | | --- | | 0.76 | | 4 (108.5) | | | 4 (108.2) | | 8.7 (<0.001) |
| 1446094_at | | zinc finger homeobox 3 | | Zfhx3 | | 0.66 | | 8 (111.3) | | | 4 (111.0) | | 4.9 (0.001) |
| 1452752_at | | leprecan 1 | | Lepre1 | | 0.59 | | 4 (118.9) | | | 4 (114.5) | | 4.9 (0.004) |
| 1446057_at | | rearranged L-myc fusion sequence | | Rlf | | 0.58 | | 4 (120.9) | | | 4 (120.7) | | 5.6 (<0.001) |
| 1450673_at | | collagen, type IX, alpha 2 | | Col9a2 | | 0.56 | | 4 (120.7) | | | 4 (120.7) | | 4.4 (0.011) |
| 1418651_at | | spermatogenesis associated 6 | | Spata6 | | 0.54 | | 4 (111.4) | | | 4 (114.5) | | 3.9 (0.057) |
| 1456557_at | | coiled-coil domain containing 30 | | Ccdc30 | | 0.49 | | 4 (119.0) | | | 4 (114.5) | | 3.7 (0.038) |
| **Prefrontal Cortex: salmon3** | | | | | | | | | | | | | |
| **Probeset** | | **Gene Title** | | **Gene Symbol** | | **Connectivity** | | **Physcial Location: chr (Mb)** | | | **eQTL Location: chr (Mb)** | | **LOD score (p-value)** |
| 1457726_at | | ribosomal protein S15A | | Rps15a | | 1.84 | | 7 (125.2) | | | 7 (124.4) | | 6.3 (0.009) |
| 1456981_at | | transmembrane channel-like gene family 7 | | Tmc7 | | 1.62 | | 7 (125.8) | | | 7 (124.4) | | 6.2 (0.019) |
| 1423819_s_at | | ADP-ribosylation factor-like 6 interacting protein 1 | | Arl6ip1 | | 1.50 | | 7 (125.3) | | | 7 (120.2) | | 3.8 (0.048) |
| 1423818_a_at | | ADP-ribosylation factor-like 6 interacting protein 1 | | Arl6ip1 | | 1.43 | | 7 (125.3) | | | 7 (124.4) | | 4.2 (0.053) |
| 1435390_at | | exoribonuclease 2 | | Eri2 | | 0.76 | | 7 (126.9) | | | 7 (126.3) | | 3.8 (0.071) |
| 1427568_a_at | | intraflagellar transport 80 homolog (Chlamydomonas) | | Ift80 | | 0.60 | | 3 (68.7) | | | 11 (4.4) | | 3.1 (0.181) |
| 1443724_at | | junctophilin 3 | | Jph3 | | 0.48 | | 8 (124.3) | | | 7 (126.3) | | 4.5 (0.022) |
| **Prefrontal Cortex: lightsteelblue1** | | | | | | | | | | | | | |
| **Probeset** | | **Gene Title** | | **Gene Symbol** | | **Connectivity** | | **Physcial Location: chr (Mb)** | | | **eQTL Location: chr (Mb)** | | **LOD score (p-value)** |
| 1451457_at | | sterol-C5-desaturase (fungal ERG3, delta-5-desaturase) homolog (S. cerevisae) | | Sc5d | | 6.65 | | 9 (42.1) | | | 9 (41.5) | | 12.6 (0.007) |
| 1447047_at | | 12 days embryo eyeball cDNA, RIKEN full-length enriched library, clone:D230004N17 product | | AK084170 | | 5.54 | | 9 (41.4) | | | 9 (41.5) | | 10.5 (0.006) |
| 1453003_at | | sortilin-related receptor, LDLR class A repeats-containing | | Sorl1 | | 4.64 | | 9 (41.8) | | | 9 (41.5) | | 6.3 (0.015) |
| 1423290_at | | hypoxia up-regulated 1 | | Hyou1 | | 4.31 | | 9 (44.2) | | | 9 (41.5) | | 6.9 (0.011) |
| 1442680_at | | neural cell adhesion molecule 1 | | Ncam1 | | 3.95 | | 9 (49.5) | | | 9 (41.5) | | 5.7 (0.009) |
| 1426865_a_at | | neural cell adhesion molecule 1 | | Ncam1 | | 3.83 | | 9 (49.3) | | | 9 (46.0) | | 7.2 (0.002) |
| 1444248_at | | reticulocalbin 2 | | Rcn2 | | 3.82 | | 9 (55.9) | | | 9 (51.5) | | 11.4 (0.001) |
| 1460390_at | | sortilin-related receptor, LDLR class A repeats-containing | | Sorl1 | | 3.79 | | 9 (41.8) | | | 9 (41.5) | | 3.9 (0.054) |
| 1418844_at | | asparagine-linked glycosylation 9 homolog (yeast, alpha 1,2 mannosyltransferase) | | Alg9 | | 3.73 | | 9 (50.6) | | | 9 (46.0) | | 6.4 (0.003) |
| 1456111_at | | family with sequence similarity 55, member D | | Fam55d | | 3.69 | | 9 (48.2) | | | 9 (46.0) | | 7.6 (0.002) |
| 1457223_at | | ELMO domain containing 1 | | Elmod1 | | 3.58 | | 9 (53.8) | | | 9 (51.5) | | 4.2 (0.079) |
| 1417474_at | | intraflagellar transport 46 homolog (Chlamydomonas) | | Ift46 | | 3.55 | | 9 (44.6) | | | 9 (41.5) | | 5.1 (0.028) |
| 1446608_at | | Casitas B-lineage lymphoma | | Cbl | | 3.41 | | 9 (44.0) | | | 9 (41.5) | | 5.4 (0.002) |
| 1451913_a_at | | hypoxia up-regulated 1 | | Hyou1 | | 3.25 | | 9 (44.2) | | | 9 (41.5) | | 5.1 (0.017) |
| 1417211_a_at | | RIKEN cDNA 1110032A03 gene | | 1110032A03Rik | | 3.00 | | 9 (50.6) | | | 11 (76.8) | | 4.8 (0.047) |
| 1436805_at | | ubiquitin associated and SH3 domain containing, B | | Ubash3b | | 2.60 | | 9 (40.8) | | | 9 (40.3) | | 8.1 (0.008) |
| 1423291_s_at | | hypoxia up-regulated 1 | | Hyou1 | | 2.46 | | 9 (44.2) | | | 9 (46.0) | | 5.1 (0.013) |
| 1448251_at | | RIKEN cDNA 9030425E11 gene | | 9030425E11Rik | | 2.09 | | 9 (40.5) | | | 9 (40.3) | | 6.7 (0.003) |
| 1448250_at | | RIKEN cDNA 9030425E11 gene | | 9030425E11Rik | | 1.68 | | 9 (40.5) | | | 9 (40.3) | | 3.8 (0.025) |
| 1439784_at | | RIKEN cDNA 2310046O06 gene | | 2310046O06Rik | | 1.58 | | 9 (57.4) | | | 9 (56.7) | | 4.3 (0.002) |
| 1459485_at | | neogenin | | Neo1 | | 1.57 | | 9 (58.8) | | | 16 (96.9) | | 3.3 (0.280) |
| 1424709_at | | sterol-C5-desaturase (fungal ERG3, delta-5-desaturase) homolog (S. cerevisae) | | Sc5d | | 1.53 | | 9 (42.1) | | | 9 (41.5) | | 4.1 (0.042) |
| 1447083_at | | 0 day neonate lung cDNA, RIKEN full-length enriched library, clone:E030013G21 product | | AK086943 | | 1.47 | | 9 (51.7) | | | 9 (56.7) | | 3.7 (0.037) |
| 1441457_at | | neuroplastin precursor | | Nptn | | 1.46 | | 9 (58.5) | | | 9 (56.7) | | 3.6 (0.046) |
| 1427437_at | | RIKEN cDNA 2610203C20 gene | | 2610203C20Rik | | 1.31 | | 9 (41.4) | | | 9 (41.5) | | 3.4 (0.007) |
| 1436467_at | | RIKEN cDNA D230004N17 gene /// hypothetical LOC100504017 | | D230004N17Rik /// LOC100504017 | | 1.14 | | 9 (41.4) | | | 4 (132.4) | | 3.3 (0.236) |
| 1423513_at | | UHRF1 (ICBP90) binding protein 1-like | | Uhrf1bp1l | | 1.01 | | 9 (58.7) | | | 9 (56.7) | | 3.6 (0.017) |
| 1444855_at | | B-cell CLL/lymphoma 9-like protein | | Bcl9l | | 0.84 | | 9 (44.3) | | | 5 (23.9) | | 3.3 (0.085) |
| 1453846_at | | chondroitin polymerizing factor 2 | | Chpf2 | | 0.84 | | 5 (24.1) | | | 9 (41.5) | | 2.8 (0.232) |
| 1451079_at | | ADP-dependent glucokinase | | Adpgk | | 0.83 | | 9 (59.1) | | | 9 (56.7) | | 2.4 (0.581) |
| 1446540_at | | kin of IRRE like 3 (Drosophila) | | Kirrel3 | | 0.82 | | 9 (34.5) | | | 9 (29.7) | | 7.6 (0.006) |
| 1423688_at | | mannosidase, alpha, class 2C, member 1 | | Man2c1 | | 0.75 | | 9 (57.0) | | | 9 (56.7) | | 2.4 (0.262) |
| 1441613_at | | Zinc finger protein 40 | | Zfp40 | | 0.72 | | 17 (23.3) | | | 4 (154.9) | | 3.0 (0.257) |
| 1434008_at | | sodium channel, type IV, beta | | Scn4b | | 0.66 | | 9 (45.0) | | | 18 (68.7) | | 2.9 (0.202) |
| 1441022_at | | ariadne ubiquitin-conjugating enzyme E2 binding protein homolog 1 (Drosophila) | | Arih1 | | 0.56 | | 9 (59.2) | | | 16 (96.9) | | 3.9 (0.067) |
| 1435580_at | | RIKEN cDNA C230081A13 gene | | C230081A13Rik | | 0.56 | | 9 (56.0) | | | 9 (51.5) | | 4.7 (0.037) |
| 1437335_x_at | | polymerase (DNA-directed), delta interacting protein 3 | | Poldip3 | | 0.47 | | 15 (83.0) | | | 4 (41.6) | | 2.2 (0.486) |
| **Prefrontal Cortex: darkorange.1** | | | | | | | | | | | | | |
| **Probeset** | | **Gene Title** | | **Gene Symbol** | | **Connectivity** | | **Physcial Location: chr (Mb)** | | | **eQTL Location: chr (Mb)** | | **LOD score (p-value)** |
| 1416658_at | | frizzled-related protein | | Frzb | | 1.13 | | 2 (80.3) | | | 2 (79.2) | | 13.4 (<0.001) |
| 1448424_at | | frizzled-related protein | | Frzb | | 1.05 | | 2 (80.3) | | | 2 (79.1) | | 11.7 (<0.001) |
| 1426904_s_at | | DnaJ (Hsp40) homolog, subfamily C, member 10 | | Dnajc10 | | 0.48 | | 2 (80.2) | | | 2 (83.3) | | 4.2 (0.031) |
| 1426905_a_at | | DnaJ (Hsp40) homolog, subfamily C, member 10 | | Dnajc10 | | 0.36 | | 2 (80.2) | | | 7 (31.5) | | 4.4 (0.023) |
| 1437904_at | | RNA binding motif protein 45 | | Rbm45 | | 0.30 | | 2 (76.2) | | | 2 (75.9) | | 15.6 (<0.001) |
| 1452230_at | | DnaJ (Hsp40) homolog, subfamily C, member 10 | | Dnajc10 | | 0.27 | | 2 (80.2) | | | 2 (83.3) | | 3.3 (0.112) |
| **Prefrontal Cortex: bisque4.1** | | | | | | | | | | | | | |
| **Probeset** | | **Gene Title** | | **Gene Symbol** | | **Connectivity** | | **Physcial Location: chr (Mb)** | | | **eQTL Location: chr (Mb)** | | **LOD score (p-value)** |
| 1443745_s_at | | dentin matrix protein 1 | | Dmp1 | | 1.31 | | 5 (104.6) | | | 9 (28.1) | | 3.4 (0.100) |
| 1443746_x_at | | dentin matrix protein 1 | | Dmp1 | | 1.10 | | 5 (104.6) | | | 2 (181.0) | | 2.3 (0.539) |
| 1426005_at | | dentin matrix protein 1 | | Dmp1 | | 1.01 | | 5 (104.6) | | | 9 (28.1) | | 3.0 (0.124) |
| 1420347_at | | palate, lung, and nasal epithelium associated | | Plunc | | 0.94 | | 2 (154.0) | | | 9 (28.1) | | 16.3 (0.052) |
| 1427050_at | | thioredoxin domain containing 16 | | Txndc16 | | 0.87 | | 14 (45.8) | | | 9 (28.1) | | 14.2 (0.034) |
| 1440908_at | | hypothetical protein D030063E12 | | D030063E12 | | 0.18 | | 15 (38.9) | | | 9 (28.1) | | 4.0 (0.055) |
| **Striatum: burlywood3** | | | | | | | | | | | | | |
| **Probeset** | | **Gene Title** | | **Gene Symbol** | | **Connectivity** | | **Physcial Location: chr (Mb)** | | | **eQTL Location: chr (Mb)** | | **LOD score (p-value)** |
| 1457726_at | | ribosomal protein S15A | | Rps15a | | 0.55 | | 7 (125.2) | | | 7 (124.4) | | 14.1 (<0.001) |
| 1424415_s_at | | spondin 1, (f-spondin) extracellular matrix protein | | Spon1 | | 0.41 | | 7 (120.9) | | | 7 (122.6) | | 10.5 (<0.001) |
| 1436541_at | | RIKEN cDNA 2310008H09 gene | | 2310008H09Rik | | 0.36 | | 7 (126.0) | | | 7 (124.4) | | 9.9 (<0.001) |
| 1451411_at | | G protein-coupled receptor, family C, group 5, member B | | Gprc5b | | 0.25 | | 7 (126.1) | | | 7 (124.4) | | 6.6 (<0.001) |
| 1441226_at | | spondin 1, (f-spondin) extracellular matrix protein | | Spon1 | | 0.23 | | 7 (121.1) | | | 7 (122.6) | | 4.6 (0.002) |

| **Table S4 Characteristics of Candidate Meta-Modules** | | | |
| --- | --- | --- | --- |
| **meta-turquoise** | | | |
| **Brain Area(s)** | **Gene Name** | **Gene Symbol** | **Physcial Location: chr (Mb)** |
| PFC | 0 day neonate lung cDNA, RIKEN full-length enriched library, clone:E030013G21 product | AK086943 | 9 (51.7) |
| NA, PFC, VTA | 12 days embryo eyeball cDNA, RIKEN full-length enriched library, clone:D230004N17 product | AK084170 | 9 (41.4) |
| PFC, VTA | ADP-dependent glucokinase | Adpgk | 9 (59.1) |
| NA, PFC | ariadne ubiquitin-conjugating enzyme E2 binding protein homolog 1 (Drosophila) | Arih1 | 9 (59.2) |
| NA, PFC, VTA | asparagine-linked glycosylation 9 homolog (yeast, alpha 1,2 mannosyltransferase) | Alg9 | 9 (50.6) |
| NA, PFC, VTA | B-cell CLL/lymphoma 9-like protein | Bcl9l | 9 (44.3) |
| NA, PFC, VTA | Casitas B-lineage lymphoma | Cbl | 9 (44.0) |
| PFC | chondroitin polymerizing factor 2 | Chpf2 | 5 (24.1) |
| VTA | decapping enzyme, scavenger | Dcps | 9 (34.9) |
| NA | dihydrolipoamide S-acetyltransferase (E2 component of pyruvate dehydrogenase complex) | Dlat | 9 (50.4) |
| NA | DIS3 mitotic control homolog (S. cerevisiae)-like | Dis3l | 9 (64.2) |
| NA | DIX domain containing 1 | Dixdc1 | 9 (50.5) |
| NA, PFC | ELMO domain containing 1 | Elmod1 | 9 (53.8) |
| NA, PFC, VTA | family with sequence similarity 55, member D | Fam55d | 9 (48.2) |
| NA | Fc receptor-like A | Fcrla | 1 (172.8) |
| NA | FXYD domain-containing ion transport regulator 2 | Fxyd2 | 9 (45.2) |
| VTA | glutamate receptor, ionotropic, kainate 4 | Grik4 | 9 (42.3) |
| NA | heat shock protein 8 /// hypothetical LOC624853 | Hspa8 /// LOC624853 | 9 (40.6) |
| NA, VTA | high mobility group 20A | Hmg20a | 9 (56.3) |
| NA, PFC, VTA | hypoxia up-regulated 1 | Hyou1 | 9 (44.2) |
| NA, PFC, VTA | intraflagellar transport 46 homolog (Chlamydomonas) | Ift46 | 9 (44.6) |
| NA | keratin 24 | Krt24 | 11 (99.1) |
| PFC, VTA | kin of IRRE like 3 (Drosophila) | Kirrel3 | 9 (34.5) |
| PFC | mannosidase, alpha, class 2C, member 1 | Man2c1 | 9 (57.0) |
| NA, PFC, VTA | neogenin | Neo1 | 9 (58.8) |
| NA, PFC, VTA | neural cell adhesion molecule 1 | Ncam1 | 9 (49.5) |
| PFC | neuroplastin precursor | Nptn | 9 (58.5) |
| NA | OAF homolog (Drosophila) | Oaf | 9 (43.0) |
| PFC | polymerase (DNA-directed), delta interacting protein 3 | Poldip3 | 15 (83.0) |
| VTA | predicted gene 5617 | Gm5617 | 9 (48.3) |
| NA | RAR-related orphan receptor alpha | Rora | 9 (68.8) |
| NA, PFC, VTA | reticulocalbin 2 | Rcn2 | 9 (55.9) |
| NA | Rho guanine nucleotide exchange factor (GEF) 12 | Arhgef12 | 9 (42.8) |
| NA | ribosomal protein S25 | Rps25 | 9 (44.2) |
| NA, PFC, VTA | RIKEN cDNA 1110032A03 gene | 1110032A03Rik | 9 (50.6) |
| NA | RIKEN cDNA 1700017B05 gene | 1700017B05Rik | 9 (57.1) |
| VTA | RIKEN cDNA 1700063D05 gene | 1700063D05Rik | 9 (41.0) |
| NA, VTA | RIKEN cDNA 2310030G06 gene | 2310030G06Rik | 9 (50.5) |
| PFC, VTA | RIKEN cDNA 2310046O06 gene | 2310046O06Rik | 9 (57.4) |
| NA, PFC, VTA | RIKEN cDNA 2610203C20 gene | 2610203C20Rik | 9 (41.4) |
| NA, PFC, VTA | RIKEN cDNA 9030425E11 gene | 9030425E11Rik | 9 (40.5) |
| PFC | RIKEN cDNA C230081A13 gene | C230081A13Rik | 9 (56.0) |
| NA, PFC, VTA | RIKEN cDNA D230004N17 gene /// hypothetical LOC100504017 | D230004N17Rik /// LOC100504017 | 9 (41.2) |
| NA, PFC, VTA | sodium channel, type IV, beta | Scn4b | 9 (45.0) |
| NA, PFC, VTA | sortilin-related receptor, LDLR class A repeats-containing | Sorl1 | 9 (41.8) |
| NA, PFC, VTA | sterol-C5-desaturase (fungal ERG3, delta-5-desaturase) homolog (S. cerevisae) | Sc5d | 9 (42.1) |
| NA, PFC, VTA | ubiquitin associated and SH3 domain containing, B | Ubash3b | 9 (40.8) |
| NA, PFC | UHRF1 (ICBP90) binding protein 1-like | Uhrf1bp1l | 9 (58.7) |
| NA | von Willebrand factor A domain containing 5A | Vwa5a | 9 (38.5) |
| PFC | Zinc finger protein 40 | Zfp40 | 17 (23.3) |
| **meta-blue** | | | |
| **Brain Area(s)** | **Gene Name** | **Gene Symbol** | **Physcial Location: chr (Mb)** |
| STR | RIKEN cDNA 2310008H09 gene | 2310008H09Rik | 7 (126.0) |
| WB | aryl hydrocarbon receptor nuclear translocator-like Gene | Arntl | 7 (120.4) |
| VTA, WB | demethyl-Q 7 | Coq7 | 7 (125.7) |
| VTA, WB | exoribonuclease 2 | Eri2 | 7 (126.9) |
| STR, VTA, WB | G protein-coupled receptor, family C, group 5, member B | Gprc5b | 7 (126.1) |
| VTA | potassium voltage-gated channel, subfamily H (eag-related), member 1 | Kcnh1 | 1 (194.0) |
| WB | pleckstrin homology domain containing, family A member 7 Gene | Plekha7 | 7 (123.3) |
| STR, VTA, WB | ribosomal protein S15A | Rps15a | 7 (125.2) |
| VTA | SMG1 homolog, phosphatidylinositol 3-kinase-related kinase (C. elegans) | Smg1 | 7 (125.3) |
| STR, VTA, WB | spondin 1, (f-spondin) extracellular matrix protein | Spon1 | 7 (120.9) |
| VTA | THUMP domain containing 1 | Thumpd1 | 7 (126.9) |
| VTA, WB | transmembrane channel-like gene family 7 | Tmc7 | 7 (125.7) |
| **meta-brown** | | | |
| **Brain Area(s)** | **Gene Name** | **Gene Symbol** | **Physcial Location: chr (Mb)** |
| NA | archaelysin family metallopeptidase 2 | Amz2 | 11 (109.3) |
| VTA | cytochrome b-561 | Cyb561 | 11 (105.8) |
| VTA | microtubule-associated protein tau | Mapt | 11 (104.1) |
| NA | frizzled homolog 2 (Drosophila) | Fzd2 | 11 (102.5) |
| NA | membrane protein, palmitoylated 3 (MAGUK p55 subfamily member 3) | Mpp3 | 11 (101.9) |
| NA | phosphatidylinositol transfer protein, cytoplasmic 1 | Pitpnc1 | 11 (107.1) |
| NA, VTA | LSM12 homolog (S. cerevisiae) | Lsm12 | 11 (102.0) |
| NA | regulator of G-protein signaling 9 | Rgs9 | 11 (109.1) |
| NA, VTA | glial fibrillary acidic protein | Gfap | 11 (102.8) |
| VTA | RIKEN cDNA 1700081L11 gene | 1700081L11Rik | 11 (104.2) |
| NA | major facilitator superfamily domain containing 9 | Mfsd9 | 1 (40.8) |

| **Table S5 Correlation Among Transcripts After Correction for cis-eQTLs** | | | | | | | | | | | | | | | | |
| --- | --- | --- | --- | --- | --- | --- | --- | --- | --- | --- | --- | --- | --- | --- | --- | --- |
|  | **Rps15a** | **Hyou1** | **Arih1** | **231003** | **Rcn2** | **Fxyd2** | **1700017** | **Lsm12** | **Dis3l** | **Hyou1** | **Chpf2** | **Alg9** | **Ubash3b** | **Sorl1** | **Lsm12** | **Thumpd1** |
|  | **1457726_at_str** | **1423290_at_na** | **1441022_at_na** | **1449357_at_na** | **1444248_at_na** | **1419378_a_at_na** | **1447063_at_na** | **1429509_at_na** | **1437737_at_na** | **1423291_s_at_pfc** | **1453846_at_pfc** | **1418844_at_pfc** | **1436805_at_pfc** | **1460390_at_pfc** | **1427998_at_vta** | **1436007_a_at_vta** |
| **Rps15a (str)** |  | 0.03 | -0.27 | -0.09 | 0.08 | -0.38 | -0.06 | -0.20 | -0.11 | 0.13 | -0.03 | -0.03 | 0.09 | -0.23 | 0.12 | 0.06 |
|  |  | (0.867) | (0.179) | (0.641) | (0.691) | (0.051) | (0.764) | (0.316) | (0.571) | (0.521) | (0.898) | (0.893) | (0.647) | (0.238) | (0.531) | (0.764) |
| **Hyou1 (na)** | 0.03 |  | -0.52 | -0.39 | -0.21 | -0.23 | -0.11 | 0.08 | -0.09 | 0.58 | -0.01 | 0.03 | 0.40 | -0.04 | -0.17 | -0.21 |
|  | (0.867) |  | (0.002) | (0.022) | (0.240) | (0.189) | (0.546) | (0.666) | (0.607) | (0.002) | (0.968) | (0.888) | (0.041) | (0.833) | (0.335) | (0.227) |
| **Arih1 (na)** | -0.27 | -0.52 |  | 0.56 | 0.18 | 0.31 | -0.24 | -0.17 | 0.50 | -0.38 | 0.13 | -0.04 | -0.08 | 0.24 | 0.03 | 0.15 |
|  | (0.179) | (0.002) |  | (0.001) | (0.298) | (0.077) | (0.169) | (0.330) | (0.003) | (0.054) | (0.534) | (0.864) | (0.709) | (0.235) | (0.884) | (0.391) |
| **2310030G06Rik (na)** | -0.09 | -0.39 | 0.56 |  | 0.17 | 0.45 | -0.13 | -0.39 | 0.53 | -0.41 | 0.07 | 0.11 | -0.03 | 0.04 | -0.10 | -0.14 |
|  | (0.641) | (0.022) | (0.001) |  | (0.332) | (0.007) | (0.461) | (0.023) | (0.001) | (0.039) | (0.733) | (0.588) | (0.878) | (0.840) | (0.588) | (0.432) |
| **Rcn2 (na)** | 0.08 | -0.21 | 0.18 | 0.17 |  | 0.14 | -0.12 | 0.15 | 0.38 | -0.18 | 0.06 | 0.01 | -0.29 | 0.05 | 0.25 | 0.24 |
|  | (0.691) | (0.240) | (0.298) | (0.332) |  | (0.434) | (0.514) | (0.395) | (0.026) | (0.377) | (0.777) | (0.955) | (0.145) | (0.810) | (0.154) | (0.170) |
| **Fxyd2 (na)** | -0.38 | -0.23 | 0.31 | 0.45 | 0.14 |  | -0.24 | 0.02 | 0.31 | 0.14 | 0.06 | 0.20 | -0.05 | -0.03 | -0.13 | 0.03 |
|  | (0.051) | (0.189) | (0.077) | (0.007) | (0.434) |  | (0.173) | (0.931) | (0.079) | (0.486) | (0.788) | (0.335) | (0.819) | (0.874) | (0.452) | (0.871) |
| **1700017B05Rik (na)** | -0.06 | -0.11 | -0.24 | -0.13 | -0.12 | -0.24 |  | -0.10 | -0.41 | 0.03 | -0.03 | -0.25 | -0.21 | -0.50 | 0.25 | -0.07 |
|  | (0.764) | (0.546) | (0.169) | (0.461) | (0.514) | (0.173) |  | (0.565) | (0.017) | (0.897) | (0.899) | (0.228) | (0.304) | (0.010) | (0.161) | (0.699) |
| **Lsm12 (na)** | -0.20 | 0.08 | -0.17 | -0.39 | 0.15 | 0.02 | -0.10 |  | 0.01 | 0.10 | -0.08 | 0.19 | -0.16 | 0.30 | 0.01 | -0.08 |
|  | (0.316) | (0.666) | (0.330) | (0.023) | (0.395) | (0.931) | (0.565) |  | (0.971) | (0.619) | (0.710) | (0.364) | (0.429) | (0.135) | (0.967) | (0.662) |
| **Dis3l (na)** | -0.11 | -0.09 | 0.50 | 0.53 | 0.38 | 0.31 | -0.41 | 0.01 |  | -0.27 | 0.36 | 0.04 | 0.11 | 0.31 | 0.05 | -0.02 |
|  | (0.571) | (0.607) | (0.003) | (0.001) | (0.026) | (0.079) | (0.017) | (0.971) |  | (0.183) | (0.068) | (0.840) | (0.594) | (0.121) | (0.775) | (0.925) |
| **Hyou1 (pfc)** | 0.13 | 0.58 | -0.38 | -0.41 | -0.18 | 0.14 | 0.03 | 0.10 | -0.27 |  | -0.06 | 0.21 | 0.39 | -0.06 | -0.04 | -0.06 |
|  | (0.521) | (0.002) | (0.054) | (0.039) | (0.377) | (0.486) | (0.897) | (0.619) | (0.183) |  | (0.751) | (0.283) | (0.047) | (0.749) | (0.858) | (0.762) |
| **Chpf2 (pfc)** | -0.03 | -0.01 | 0.13 | 0.07 | 0.06 | 0.06 | -0.03 | -0.08 | 0.36 | -0.06 |  | -0.36 | 0.12 | -0.20 | -0.10 | 0.07 |
|  | (0.898) | (0.968) | (0.534) | (0.733) | (0.777) | (0.788) | (0.899) | (0.710) | (0.068) | (0.751) |  | (0.067) | (0.560) | (0.315) | (0.606) | (0.731) |
| **Alg9 (pfc)** | -0.03 | 0.03 | -0.04 | 0.11 | 0.01 | 0.20 | -0.25 | 0.19 | 0.04 | 0.21 | -0.36 |  | -0.09 | 0.49 | -0.07 | -0.20 |
|  | (0.893) | (0.888) | (0.864) | (0.588) | (0.955) | (0.335) | (0.228) | (0.364) | (0.840) | (0.283) | (0.067) |  | (0.651) | (0.010) | (0.738) | (0.307) |
| **Ubash3b (pfc)** | 0.09 | 0.40 | -0.08 | -0.03 | -0.29 | -0.05 | -0.21 | -0.16 | 0.11 | 0.39 | 0.12 | -0.09 |  | -0.11 | 0.03 | -0.10 |
|  | (0.647) | (0.041) | (0.709) | (0.878) | (0.145) | (0.819) | (0.304) | (0.429) | (0.594) | (0.047) | (0.560) | (0.651) |  | (0.578) | (0.869) | (0.604) |
| **Sorl1 (pfc)** | -0.23 | -0.04 | 0.24 | 0.04 | 0.05 | -0.03 | -0.50 | 0.30 | 0.31 | -0.06 | -0.20 | 0.49 | -0.11 |  | 0.20 | 0.24 |
|  | (0.238) | (0.833) | (0.235) | (0.840) | (0.810) | (0.874) | (0.010) | (0.135) | (0.121) | (0.749) | (0.315) | (0.010) | (0.578) |  | (0.316) | (0.232) |
| **Lsm12 (vta)** | 0.12 | -0.17 | 0.03 | -0.10 | 0.25 | -0.13 | 0.25 | 0.01 | 0.05 | -0.04 | -0.10 | -0.07 | 0.03 | 0.20 |  | 0.42 |
|  | (0.531) | (0.335) | (0.884) | (0.588) | (0.154) | (0.452) | (0.161) | (0.967) | (0.775) | (0.858) | (0.606) | (0.738) | (0.869) | (0.316) |  | (0.011) |
| **Thumpd1 (vta)** | 0.06 | -0.21 | 0.15 | -0.14 | 0.24 | 0.03 | -0.07 | -0.08 | -0.02 | -0.06 | 0.07 | -0.20 | -0.10 | 0.24 | 0.42 |  |
|  | (0.764) | (0.227) | (0.391) | (0.432) | (0.170) | (0.871) | (0.699) | (0.662) | (0.925) | (0.762) | (0.731) | (0.307) | (0.604) | (0.232) | (0.011) |  |

Partial correlation coefficients (significant p-values (p<0.05) and correlation coefficients are shaded) were calculated among probesets in the meta-modules, accounting for the most proximal marker to the probeset (see text).
